# DNA methylome-wide association study of genetic risk for depression implicates antigen processing and immune responses

**DOI:** 10.1186/s13073-022-01039-5

**Published:** 2022-03-31

**Authors:** Xueyi Shen, Doretta Caramaschi, Mark J. Adams, Rosie M. Walker, Josine L. Min, Alex Kwong, Gibran Hemani, Genetics of DNA Methylation Consortium, Miruna C. Barbu, Heather C. Whalley, Sarah E. Harris, Ian J. Deary, Stewart W. Morris, Simon R. Cox, Caroline L. Relton, Riccardo E. Marioni, Kathryn L. Evans, Andrew M. McIntosh

**Affiliations:** 1 Division of Psychiatry, University of Edinburgh, Royal Edinburgh Hospital, Morningside Park, Edinburgh, EH10 5HF UK; 2 College of Life and Environmental Sciences, Psychology, University of Exeter, Exeter, UK; 3 Medical Research Council Integrative Epidemiology Unit, Bristol Medical School, Population Health Sciences, University of Bristol, Bristol, UK; 4 Centre for Clinical Brain Sciences, Chancellor’s Building, 49 Little France Crescent, Edinburgh BioQuarter, Edinburgh, UK; 5 Lothian Birth Cohorts group, Department of Psychology, The University of Edinburgh, Edinburgh, UK; 6 Centre for Genomic and Experimental Medicine, Institute of Genetics and Cancer, University of Edinburgh, Edinburgh, UK

**Keywords:** DNA methylation, Polygenic risk score, Depression, Methylome-wide association study, Replication, Mendelian randomisation

## Abstract

**Background:**

Depression is a disabling and highly prevalent condition where genetic and epigenetic, such as DNA methylation (DNAm), differences contribute to disease risk. DNA methylation is influenced by genetic variation but the association between polygenic risk of depression and DNA methylation is unknown.

**Methods:**

We investigated the association between polygenic risk scores (PRS) for depression and DNAm by conducting a methylome-wide association study (MWAS) in Generation Scotland (*N* = 8898, mean age = 49.8 years) with replication in the Lothian Birth Cohorts of 1921 and 1936 and adults in the Avon Longitudinal Study of Parents and Children (ALSPAC) (*N*_combined_ = 2049, mean age = 79.1, 69.6 and 47.2 years, respectively). We also conducted a replication MWAS in the ALSPAC children (*N* = 423, mean age = 17.1 years). Gene ontology analysis was conducted for the cytosine-guanine dinucleotide (CpG) probes significantly associated with depression PRS, followed by Mendelian randomisation (MR) analysis to infer the causal relationship between depression and DNAm.

**Results:**

Widespread associations (*N*_CpG_ = 71, *p*_Bonferroni_ < 0.05, *p* < 6.3 × 10^−8^) were found between PRS constructed using genetic risk variants for depression and DNAm in CpG probes that localised to genes involved in immune responses and neural development. The effect sizes for the significant associations were highly correlated between the discovery and replication samples in adults (*r* = 0.79) and in adolescents (*r* = 0.82). Gene Ontology analysis showed that significant CpG probes are enriched in immunological processes in the human leukocyte antigen system. Additional MWAS was conducted for each lead genetic risk variant. Over 47.9% of the independent genetic risk variants included in the PRS showed associations with DNAm in CpG probes located in both the same (*cis)* and distal *(trans)* locations to the genetic loci (*p*_Bonferroni_ < 0.045). Subsequent MR analysis showed that there are a greater number of causal effects found from DNAm to depression than vice versa (DNAm to depression: *p*_FDR_ ranged from 0.024 to 7.45 × 10^−30^; depression to DNAm: *p*_FDR_ ranged from 0.028 to 0.003).

**Conclusions:**

PRS for depression, especially those constructed from genome-wide significant genetic risk variants, showed methylome-wide differences associated with immune responses. Findings from MR analysis provided evidence for causal effect of DNAm to depression.

**Supplementary Information:**

The online version contains supplementary material available at 10.1186/s13073-022-01039-5.

## Background

Depression is a highly prevalent condition and a leading cause of global disability [1], for which the underlying biological mechanisms are unclear. Genetic factors account for a substantial proportion of differences in liability to depression, which has a twin-based heritability of approximately 37% and with common genetic variants capturing around 6–10% of phenotypic variance [2, 3]. Recent genome-wide association studies (GWAS) have identified specific genetic risk variants for depression that implicate regional brain alterations [4, 5]. Polygenic risk scores (PRS) derived from the results of GWAS studies, have been widely used to estimate additive genetic risk [6]. PRS is the sum of risk alleles, weighted by the effect sizes of an independent GWAS [7]. It provides a means to identify traits that share their genetic architecture with depression, which may help to prioritise factors of biological and mechanistic relevance for the disorder [8].

DNA methylation (DNAm) at cytosine-guanine dinucleotides (CpG) sites is one of the most studied epigenetic markers and there is growing evidence of its role in understanding depression [9]. DNAm risk scores have been developed from the results of DNA methylome-wide association studies (MWAS; also widely referred to as epigenome-wide association studies or EWAS in contemporary literature) [9]. These can be used to predict prevalent depression in independent samples, and chronic depression that requires long-term treatment [10]. DNAm is influenced by both genetic and environmental factors [9, 11] and, in blood tissue, it has a mean heritability of 19% across the methylome [12] with ~7% of its variance captured by common genetic variants [12]. For the highly heritable DNAm probes, genetic effects are consistent across tissues [13] and developmental stages [12]. Genetic risk variants for diseases (e.g. schizophrenia) have been found enriched in DNAm variation [14–16]. Associations between genetic risk and epigenetic changes can enrich our understanding of the functional composition of genetic risk loci, and thus inform the mechanisms that lead to the onset of depression [17, 18]. However, systematic examination of the molecular genetic associations between genetic risk of depression and DNAm has not, to the best of our knowledge, been conducted.

In the present study, we aim to investigate the association between PRS for depression and genome-wide DNA methylation. MWAS were conducted on four cohorts: Generation Scotland: Scottish Family Health Study (GS, discovery sample, *N* = 8898) [19, 20], the Lothian Birth Cohort (LBC1921) [21, 22], the Lothian Birth Cohort 1936 (LBC1936) [21, 22], Avon Longitudinal Study of Parents and Children (ALSPAC) adults (adult replication sample, combined *N* = 2049) and ALSPAC children for replication (adolescent replication sample, *N* = 423) [23, 24]. Mendelian randomisation (MR) was used to test for causal associations between DNAm and depression using data from the Genetics of DNA Methylation Consortium (GoDMC) (*N* = 25,561) and GS.

## Methods

### Sample descriptions

#### Generation Scotland: Scottish Family Health Study (GS)

GS is a family-based population cohort with over 24,000 participants [19, 20] set up to identify the causes of common complex disorders, such as depression. DNAm data and genetic data were both collected, processed and quality-checked for 8898 people (mean age = 49.8 years, SD of age = 13.7 years, 40.90% were men) in two sets. Sample sizes for set 1 and set 2 were 4757 (mean age = 48.5 years, SD of age = 14.0 years, 38.5% were men) and 4141 (mean age = 51.4 years, SD of age = 13.2 years, 43.66% were men), respectively. Written informed consent was obtained for all participants. The study was approved by the NHS Tayside Research Ethics committee (05/s1401/89).

#### Lothian Birth Cohort (LBC)1921 and LBC1936

Participants from LBC1921 and LBC1936 [21, 22] were born in 1921 and 1936. Almost all lived in the Edinburgh and surrounding Lothian area when recruited. They are a mostly healthy, community-dwelling sample of men and women. The sample used in the current analysis included 1330 participants from both cohorts combined with genetic and DNAm data (LBC1921: mean age = 79.1 years, SD of age = 0.6, 39.7% were men; LBC1936: mean age = 69.6 years, SD of age = 0.8, 50.6% were men; all participants were unrelated). Written informed consent was obtained from all participants. Ethics permission for LBC1921 was obtained from the Lothian Research Ethics Committee (LREC/1998/4/183). Ethics permission for LBC1936 was obtained from the Multi-Centre Research Ethics Committee for Scotland (MREC/01/0/56) and the Lothian Research Ethics Committee (LREC/2003/2/29) [25, 26].

#### Avon Longitudinal Study of Parents and Children (ALSPAC)

ALSPAC is an ongoing longitudinal population-based study that recruited pregnant women residing in Avon (South-West of England) with expected delivery dates between 1st April 1991 and 31st December 1992 [23, 24]. The cohort consists of 13,761 mothers and their partners, and their 14,901 children, now young adults [27]. The study website contains details of all the data that is available through a fully searchable data dictionary and variable search tool (http://www.bristol.ac.uk/alspac/researchers/our-data/). Ethical approval for the study was obtained from the ALSPAC Ethics and Law Committee and the Local Research Ethics Committees. A subsample of 719 unrelated mothers with DNAm data (mean age = 47.2 years, SD of age = 4.6) were included in the replication study [28]. Supplementary analyses were also conducted on a younger subsample with DNAm consisting of 423 young people (mean age = 17.1 years, SD of age = 1.1 and 41% were boys). Details of the selection of participants for these subsamples are in the study by Relton et al. [28]. Consent for biological samples has been collected in accordance with the Human Tissue Act (2004).

### Genotyping and imputation

Detailed information on the quality control and genotyping methods for GS [19], LBC1921, LBC1936 [29] and ALSPAC [30] has been previously published and is described briefly below. Analyses were conducted on European participants.

#### GS

Each sample was genotyped using the IlluminaHumanOmniExpressExome-8v1.0 BeadChip (48.8%) or Illumina HumanOmniExpressExome-8 v1.2 BeadChip (51.2%) with Infinium chemistry [31]. Quality control included removing participants with genotyping call rate <98%, SNP removal of those with a minor allele frequency (MAF) <1%, call rate <98%, Hardy-Weinberg equilibrium (HWE) *p*-value <5 × 10^−6^. Imputation was performed using the Sanger Imputation server with the Haplotype Reference Consortium reference panel (HRC.r1-1). SNPs with an information metric [32] (INFO score) <0.8 were removed from the analysis.

#### LBC1921 and LBC1936

Genotyping was performed using the Illumina610-Quadv1 chip (Illumina, Inc., San Diego, CA, USA). Participants were excluded with a call rate <95%. SNPs were removed if MAF <5%, call rate <98%, HWE *p*-value <0.001. Imputation and quality control based on INFO score were the same as GS.

#### ALSPAC

Genotyping arrays used were the Illumina Human660W-quad chip for mothers and Illumina HumanHap550-quad chip for children. SNPs with missingness >0.05, HWE *p*-value <1 × 10^−6^ and MAF <0.01 were excluded. The above quality control steps were conducted on the entire genotyped sample. Imputation and quality control based on INFO score were consistent with similar procedures used in GS.

### Polygenic profiling

PRS of depression were calculated using PRSice-2 [7] for GS, LBC1921, LBC1936 and ALSPAC separately, using the summary statistics of a genome-wide meta-analysis of depression by Howard et al. [33] excluding individuals from GS previously included in that GWAS meta-analysis. The summary statistics are available at the URL: https://datashare.ed.ac.uk/handle/10283/3203 [33]. Nine *p*-value cut-offs were used for thresholding SNPs in the summary statistics (pT): 1, 0.5, 0.1, 0.05, 0.01, 1 × 10^−3^, 1 × 10^−4^, 1 × 10^−5^ and 5 × 10^−8^ for clumping and thresholding. Each set of SNPs was used to generate a depression-PRS in GS. A separate PRS was generated using the lead genetic risk variants or their closest proxies (in LD *r*^2^>0.1) reported in the GWAS by Howard et al*.* [33] for supplementary analysis. Details of the PRS profiling procedures and validation in the GS can be found elsewhere [33] (also see Additional file 1: Supplementary methods and Additional file 1: Tables S1-S2).

Subsequently, using the lead risk variants reported by Howard et al. [33], we tested for individual SNP-CpG associations in GS. Lead risk variants were selected by extracting the most significant proxy SNPs (*p* < 5 × 10^−8^) in linkage disequilibrium (LD *R*^2^ > 0.01) with the lead variants reported in the Howard et al. study [4]. A total of 96 SNPs were available and thus selected as leading risk variants for further analysis.

### DNAm data

#### GS

Genome-wide DNAm data was obtained from whole-blood samples using the Illumina Infinium Methylation EPIC array (https://emea.support.illumina.com/array/array_kits/infinium-methylationepic-beadchip-kit.html). Data processing was performed separately for each set. Quality control (QC) and normalisation were conducted using R packages ‘ShinyMethyl’ (version 1.28.0) [34], ‘watermelon’ (version 1.36.0) [35] and ‘meffil’ (version 1.1.1) [36]. Details of the protocol are described elsewhere [37]. In summary, quality control procedures removed probes if there was an outlying log median methylated signal intensity against unmethylated signal for each array, or a bead count <3 in ≥5 % of the total probe sample, or a detection *p*-value >0.05 for set 1 and *p*-value >0.01 for set 2 in ≥0.5% of the total sample in each respective set. Cross-hybridising probes that map to genetic variants at MAF >0.05 and polymorphic probes were removed [38]. Samples were excluded if sex prediction from methylation data was inconsistent with self-reported data, or a detection *p*-value >0.05 for set 1 and *p*-value >0.01 for set 2 found in >1% of the overall probes for each set respectively. The data was then normalised using the ‘dasen’ method from the ‘waterRmelon’ R package (version 1.36.0).

The raw intensities were then transformed into *M*-values by log-transforming the proportional methylation intensity [39]. The *M*-values were corrected using a linear-mixed model, controlling for relatedness using the GCTA-estimated genetic relationship matrix [40] for set 1. This step was omitted for set 2 as all participants were unrelated within the set and to set 1. The residualised *M*-values for 769,526 autosomal CpG probes were then used for further analysis.

#### LBC1921 and LBC1936

Genome-wide DNAm data was obtained from blood sample using the HumanMethylation450K array (https://emea.illumina.com/content/dam/illumina-marketing/documents/products/datasheets/datasheet_humanmethylation450.pdf) [41, 42]. Quality control and normalisation were performed using the ‘minfi’ R package (version 1.38.0) [41]. Probes with low call rate (<95%), outlying *M*-values (>3 SD from mean) or identified as cross-hybridising and polymorphic were removed [43]. Participants with insufficient cell count information were excluded from analysis.

All participants in LBC1921 and LBC1936 with methylation data were unrelated. *M*-value transformation were conducted consistently with the GS sample. Data for 409,319 CpG probes were retained for further analysis.

#### ALSPAC

Illumina Infinium HumanMethylation450 Beadchip arrays were used for measuring genome-wide DNAm data from peripheral blood samples [28]. The R package ‘meffil’ (version 1.1.1) was used for pre-processing, quality control and normalisation as previously reported [36]. Further removal of probes was conducted based on background detection (*p* > 0.05) and if they reached beyond the 3 times inter-quantile range from 25 to 75% or identified as cross-hybridising or polymorphic [43]. Related (IBD >0.1) participants were not included in the analyses [30].

*M*-value transformation was conducted. In total, 481,600 and 449,595 CpG probes remained for analysis on ALSPAC adults and children, respectively.

### Statistical models for MWAS

A discovery MWAS was initially conducted in GS. Two separate analyses were conducted on sets 1 and 2, and the final summary statistics were obtained by meta-analysing the two sets of results using METAL (version released in 2011) [44]. We used the default analysis scheme without genomic control correction (genomic inflation factors reported in the Additional file 1: Supplementary methods). *P*-values for the meta-analysis were obtained from a fixed-effect inverse-variance model. A sensitivity analysis was conducted on the unrelated participants in GS (methods and results reported in Additional file 1: Supplementary methods). A replication analysis on adults was then conducted on the total sample from LBC1921, LBC1936 and ALSPAC adults. Replication MWAS was first conducted separately for each cohort and then meta-analysed using the same parameters for the discovery analysis. Finally, an additional replication MWAS was conducted on ALSPAC children. All analyses were conducted using R (version 3.5.1) under Linux environment.

Linear regression was used to test the associations between depression-PRS and for each CpG using the R package ‘limma’ [45] (version 3.48.0) for GS, LBC1921 and LBC1936. The ‘lmFit’ function was first implemented to test the association for each CpG. The inference statistics of each linear model was then adjusted using the ‘eBayes’ function, by which an empirical Bayes method was used to adjust for gene-wise variance using a shrinkage factor. Moderated *t*-statistics and *p*-values were produced by this step. In ALSPAC, the analyses were conducted using the ‘meffil’ (version 1.1.1) R package, using the ‘sva’ option [36].

Self-reported smoking status, smoking pack years, DNAm-estimated white-blood cell proportions (CD8+T, CD4+T, natural killer cells, B cells and granulocytes) [37], batch, the first 20 principal component derived from the *M*-values, the first 10 principal components derived from the imputed genetic data, age and sex were included as covariates for the discovery methylome-wide association analysis (see Additional file 1: Supplementary Methods and Additional file 1: Table S3 for details). Where possible, the same covariates were used in the replication analyses, although only smoking status (and not pack years) was available in LBC 1921, LBC 1936 and ALSPAC. Details for all the covariates included in the replication analysis can be found in the Additional file 1: Supplementary methods. MWAS were conducted for the nine depression-PRS scores separately. *P*-values were Bonferroni-corrected (*p*-value threshold = 6.5 × 10^−8^ for EPIC array used in GS, 1.1 × 10^−7^ for 450k array used for replication analysis in LBC1921, LBC1936 and ALSPAC adults, and 1.1×10^−7^ for replication in ALSPAC children). Standardised regression coefficients are reported as effect sizes. For the significant CpG probes, gene symbol annotation and UCSC classification of CpG Island positions were acquired from the ‘UCSC_RefGene_Name’ and ‘Relation_to_Island’ columns, respectively, from the annotation object generated by the ‘IlluminaHumanMethylationEPICanno.ilm10b4.hg19’ R package (version 3.13) [46].

Individual SNP-CpG DNAm association tests were also performed, using the same covariates and *p*-value corrections as used in the PRS association analyses.

### Gene ontology analysis

Gene ontology analysis was conducted on the MWAS results from GS using the ‘gometh’ function in R package missMethyl [47]. Default settings were used, which include correction for the number of probes per gene. CpGs that showed significant association with depression-PRS at pT < 5 × 10^−8^ in the discovery analysis were selected as CpGs of interest, ‘EPIC’ was chosen for array type and all CpGs included in the analysis were used as the background list. Analyses were conducted on the Gene Ontology (GO) terms and Kyoto Encyclopedia of Genes and Genomes (KEGG) pathways separately, by specifying the ‘collection’ parameter as ‘GO’ and ‘KEGG’, respectively. FDR correction was applied for all analyses.

### Colocalisation analysis

We used Howard et al.’s depression GWAS [4] for depression-associated SNPs and GoDMC summary statistics for methylation quantitative trait loci (mQTL) analysis with PGC studies and GS study removed, which resulted in 32 remaining studies imputed to the 1000 Genome reference panel [13]. We used the package ‘gwasglue’ (version 0.0.0.9000, https://mrcieu.github.io/gwasglue/) [48] to extract SNPs that were ± 1 Mb of each of the 102 genome-wide significant, lead SNPs identified in Howard et al. [33] and then extracted the same SNPs within those regions from the GoDMC mQTL analysis. We used the coloc.abf function with default parameters in the ‘coloc’ package in R (version 5.1.0) [49] to perform colocalisation analysis for each SNP association. The method tests for five mutually exclusive scenarios in a genetic region: H_0_: there exist no causal variants for either trait; H_1_: there exists a causal variant for trait one only; H_2_: there exists a causal variant for trait two only; H_3_: there exist two distinct causal variants, one for each trait; and H_4_: there exists a single causal variant common to both traits.

### MR

Three MR methods, inverse-variance weighted (IVW), weighted median (WM) and MR-Egger, were used to test for causal effects between DNAm and depression using the ‘TwoSampleMR’ R package (version 0.5.6) [50, 51].

GWAS summary statistics for DNAm were from GoDMC and GS. mQTL summary statistics from GoDMC included 32 cohorts with 25,561 participants from European ancestry [13]. The summary statistics were computed using a two-phased design. First, every study performs a full analysis of all candidate mQTL associations, returning only associations at a threshold of *p* < 1 × 10^−5^. All candidate mQTL associations at *p* < 1 × 10^−5^ are combined to create a unique ‘candidate list’ of mQTL associations. The candidate list (*n* = 120,212,413) is then sent back to all cohorts, and the association estimates are obtained for every mQTL association on the candidate list. Candidate mQTL associations were meta-analysed using fixed-effect inverse-variance method. Details of the database can be found elsewhere [13]. mQTL summary statistics from GS (*N* = 8898) included a full set of all SNPs with no *p*-value thresholding. Summary mQTL statistics from GS were generated using the OmicS-data-based Complex Trait Analysis package (https://cnsgenomics.com/software/osca/#eQTL/mQTLAnalysis) [52]. Covariates were consistent with the MWAS for depression-PRS discovery analysis. Further details of the mQTL analysis can be found in the Additional file 1: Supplementary methods.

Summary statistics for depression GWAS by Howard et al. [33] were used. A total of 807,553 unrelated, European participants were included in the analysis. Details for the study can be found elsewhere [33].

GoDMC, GS and depression GWAS samples were mutually exclusive. Individual cohorts that overlapped with the Howard et al. depression GWAS and GS were removed from the GoDMC mQTL meta-analysis. Depression GWAS summary statistics from Howard et al*.* (2019) were calculated excluding GS participants [33]. See Additional file 1: Supplementary methods for details.

First, we used mQTL summary statistics from GoDMC to identify causal effects from DNAm to depression. Second, we used full mQTL summary statistics from GS to replicate the findings and to test the causal effect to DNAm and depression bi-directionally. Finally, in contrast to the univariable MR analyses (that is, each risk-outcome pair tested separately), a multi-variable MR analysis was conducted to test for direct causal associations from DNAm at multiple CpGs to depression, using the mQTL data from GS. Using this method, all CpG probes where there was evidence of a potential causal effect on depression were entered into the two-sample MR analysis simultaneously, in order to prioritise SNPs that showed the strongest independent casual associations with depression.

Exposures were selected from CpG probes found significantly associated with depression-PRS generated using the p-threshold = 5 × 10^−8^. The probes were further removed from analyses if they did not present in the GoDMC/GS mQTL data or had <5 independent genetic instruments overlapping with the outcome summary statistics. In result, 15 probes were selected for final analyses.

Scripts for all the above analyses can be found in the GitHub repository: https://github.com/xshen796/MDD_PRS_MWAS [53]. A detailed summary for the directories of each individual analysis can be found in the URL: https://github.com/xshen796/MDD_PRS_MWAS/wiki.

## Results

### Discovery MWAS of depression-PRS in GS

#### MWAS with depression-PRS at all p-thresholds

**Fig. 1:**
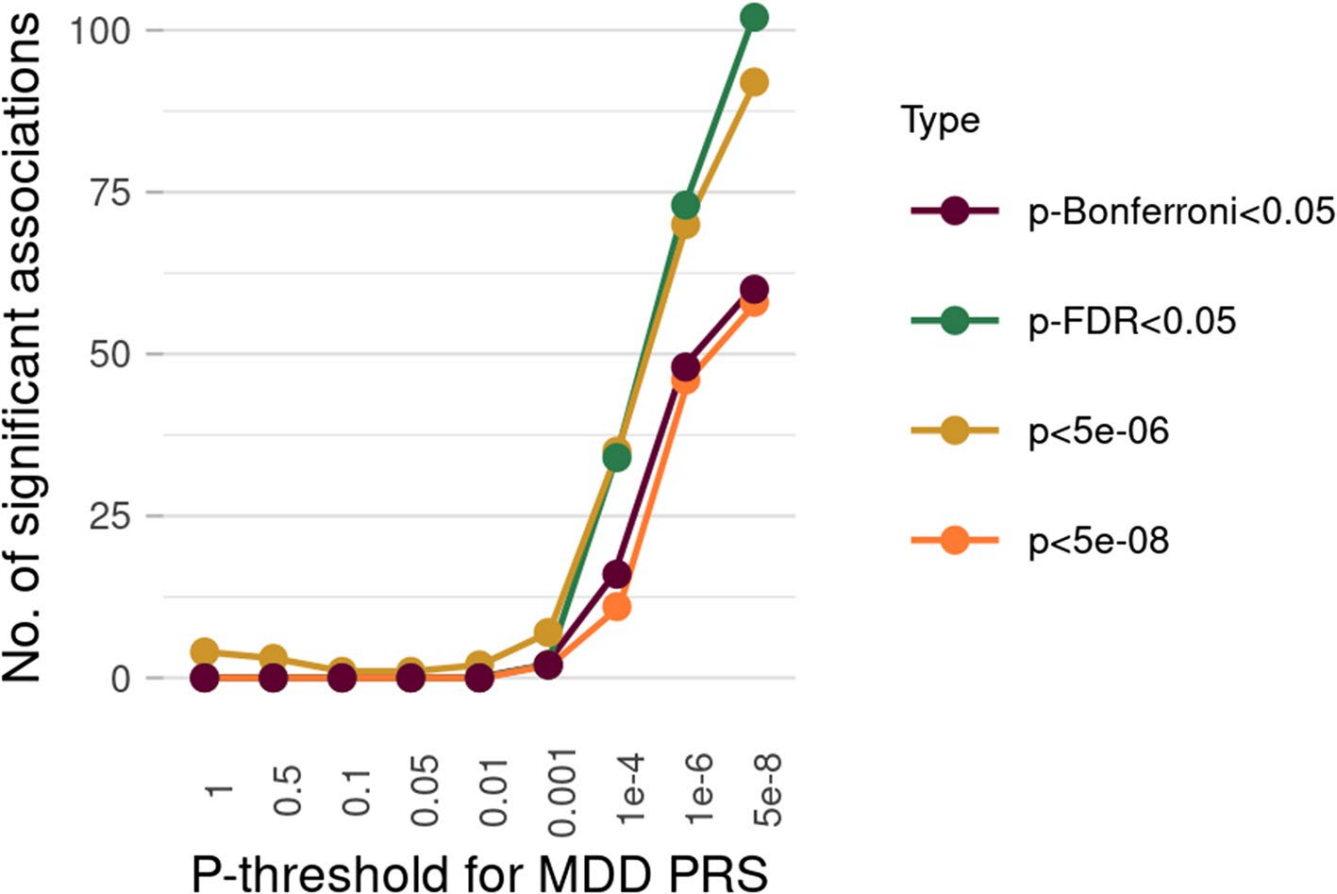
Number of CpG probes associated with polygenic risk scores (PRS) at nine different p-thresholds (pTs) for discovery analysis. *X*-axis represents the pTs used for generating PRS. *Y*-axis shows the number of probes significantly associated with the given PRS. The four different lines represent four types of methods to define significance

There were 71 CpG probes significantly associated with depression-PRS with p-threshold (pT) at 5 × 10^−8^ (*p* < 6.3 × 10^−8^ to reach significance after Bonferroni correction). In contrast to many other studies that use polygenic risk profiling at different thresholds to predict depression [54], both the number of significant associations and the effect sizes decreased as PRSs were calculated at increasingly lenient thresholds (Fig. 1). For pT of 1 × 10^−6^, 29 CpGs were associated with depression-PRS (Additional file 1: Fig. S1). No significant associations were found for PRS using *p*-value thresholds greater than or equal to 1 × 10^−4^. Quantile-quantile plot and statistics for genomic inflation factors (ranged from 0.960 to 0.970) can be found in Additional file 1: Fig. S2 and Table S4. Results using the depression-PRS calculated using only genome-wide significant variants are presented below.

#### DNAm association with depression-PRS at a GWAS p-value association threshold of 5 × 10^−8^

**Fig. 2:**
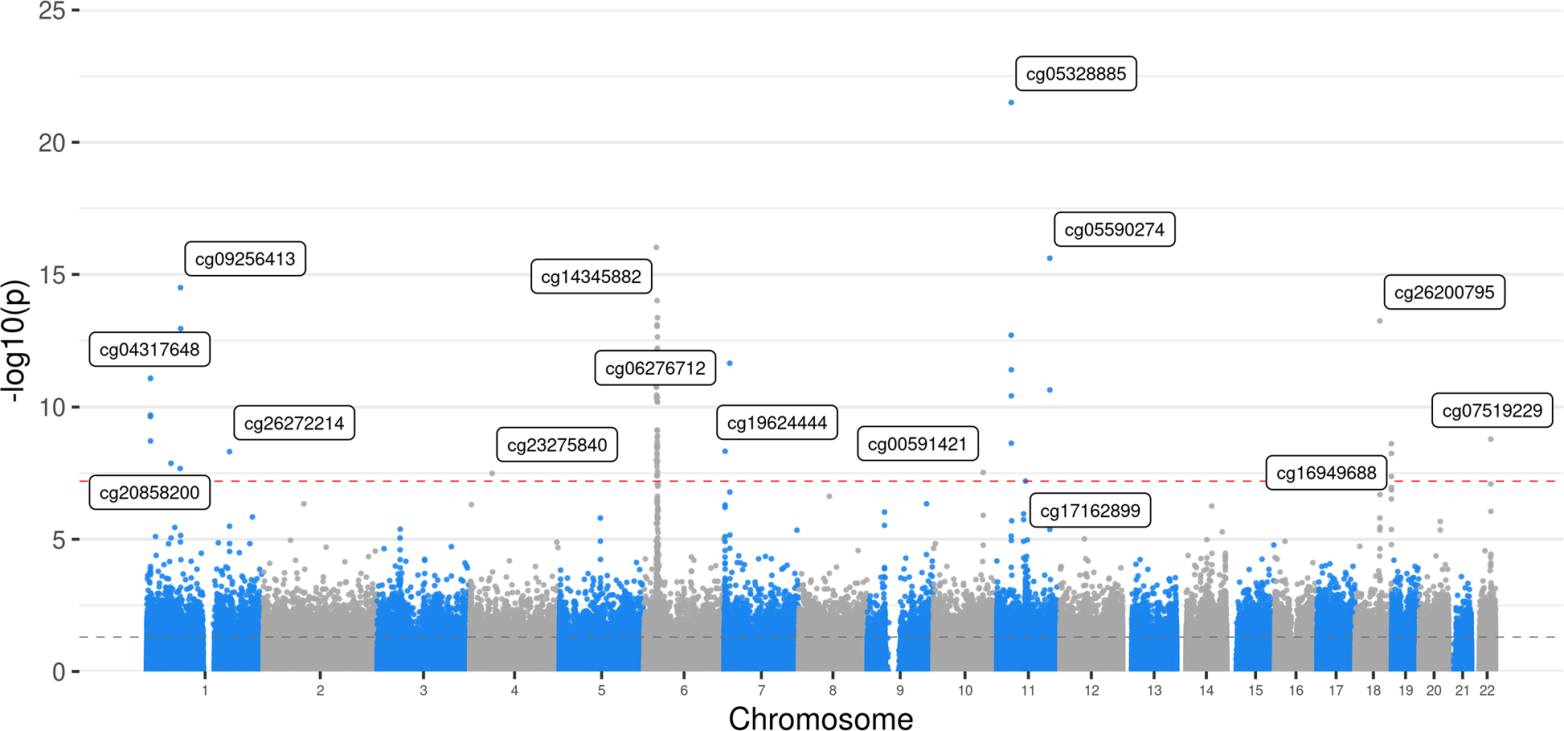
Manhattan plot for the discovery methylome-wide association study (MWAS) for PRS of pT at 5 × 10^−8^ in Generation Scotland (GS). Each dot represents a CpG probe. *X*-axis represents the relative position of the probes in the genome. *Y*-axis represents −log10-transformed *p*-values. The red dashed line represents the significance threshold for Bonferroni correction

The most significant associations of DNAm with depression-PRS were found in the major histocompatibility complex (MHC) region (25–35 Mb on Chromosome 6, Fig. 2), with 45/71 (63.4%) of significant associations within this region (*p*_Bonferroni_ ranged from 0.03 to 7.28 × 10^−11^). The top ten probes that showed the greatest associations are listed in Additional file 1: Table S5 (all *p*_Bonferroni_ < 8.62 × 10^−8^). After pruning (*r* < 0.1 for at least two nearest probes, window = 3 Mb), the top CpG probe identified within the MHC region was cg14345882 (all p_Bonferroni_ = 7.28 × 10^−11^). UCSC gene database annotation shows genes that are nearest to the significant probes in the MHC region are, for example, *TRIM27, HIST1H2AI* and *BTN3A2*. See Tables 1 and 2, Additional file 1: Table S5 and Additional file 2: Table S10.

**Table 1:** Results for gene ontology (GO) analysis for the MWAS on PRS at pT 5 × 10^−8^. Analyses were conducted separately for including and excluding the MHC region. Top ten GO terms are listed in the table. BP=biological process, CC=cellular component and MF=molecular function

	GO ID	Ontology	Term	N	DE	***P***_**DE**_	***P***_**FDR**_
With MHC region	GO:0072643	BP	Interferon-gamma secretion	22	2	2.24E−04	1
	GO:0003050	BP	Regulation of systemic arterial blood pressure by atrial natriuretic peptide	2	1	2.64E−03	1
	GO:0021691	BP	Cerebellar Purkinje cell layer maturation	2	1	3.27E−03	1
	GO:0021590	BP	Cerebellum maturation	3	1	3.64E−03	1
	GO:0021699	BP	Cerebellar cortex maturation	3	1	3.64E−03	1
	GO:1902412	BP	Regulation of mitotic cytokinesis	6	1	3.88E−03	1
	GO:0032609	BP	Interferon-gamma production	105	2	4.06E−03	1
	GO:0072686	CC	Mitotic spindle	103	2	4.94E−03	1
	GO:0046340	BP	Diacylglycerol catabolic process	4	1	5.24E−03	1
	GO:0048408	MF	Epidermal growth factor binding	4	1	5.99E−03	1
Without MHC region	GO:0072686	CC	Mitotic spindle	101	2	2.14E−03	1
	GO:0003050	BP	Regulation of systemic arterial blood pressure by atrial natriuretic peptide	2	1	2.30E−03	1
	GO:1902412	BP	Regulation of mitotic cytokinesis	6	1	2.31E−03	1
	GO:0021691	BP	Cerebellar Purkinje cell layer maturation	2	1	2.45E−03	1
	GO:0021590	BP	Cerebellum maturation	3	1	2.56E−03	1
	GO:0021699	BP	Cerebellar cortex maturation	3	1	2.56E−03	1
	GO:0046340	BP	Diacylglycerol catabolic process	4	1	4.06E−03	1
	GO:0051315	BP	Attachment of mitotic spindle microtubules to kinetochore	13	1	4.23E−03	1
	GO:0021578	BP	Hindbrain maturation	6	1	4.40E−03	1
	GO:0021626	BP	Central nervous system maturation	7	1	4.51E−03	1

**Table 2:** Results for pathway analysis for the MWAS on PRS at pT 5 × 10^−8^. Analyses were conducted separately for including and excluding the MHC region. Top ten Kyoto Encyclopedia of Genes and Genomes (KEGG) pathways are listed in the table

	KEGG pathway ID	Description	N	DE	***P***	***P***_**FDR**_
With MHC region	path:hsa05322	Systemic lupus erythematosus	112	1.5	0.093896415	1
	path:hsa04914	Progesterone-mediated oocyte maturation	88	1	0.10085395	1
	path:hsa04110	Cell cycle	123	1	0.110965189	1
	path:hsa04217	Necroptosis	147	1	0.116705111	1
	path:hsa04114	Oocyte meiosis	117	1	0.118584815	1
	path:hsa04925	Aldosterone synthesis and secretion	95	1	0.137824504	1
	path:hsa04613	Neutrophil extracellular trap formation	159	1.5	0.148531242	1
	path:hsa04514	Cell adhesion molecules	136	1	0.156764027	1
	path:hsa04723	Retrograde endocannabinoid signalling	134	1	0.157693357	1
	path:hsa05034	Alcoholism	163	1.5	0.162889755	1
Without MHC region	path:hsa04914	Progesterone-mediated oocyte maturation	88	1	0.077051	1
	path:hsa04110	Cell cycle	123	1	0.079099	1
	path:hsa04114	Oocyte meiosis	116	1	0.082834	1
	path:hsa04514	Cell adhesion molecules	118	1	0.098456	1
	path:hsa04925	Aldosterone synthesis and secretion	92	1	0.104019	1
	path:hsa04723	Retrograde endocannabinoid signalling	133	1	0.113819	1
	path:hsa05203	Viral carcinogenesis	152	1	0.127128	1
	path:hsa05166	Human T-cell leukaemia virus 1 infection	193	1	0.1555	1
	path:hsa04662	B cell receptor signalling pathway	76	0	1	1
	path:hsa05224	Breast cancer	143	0	1	1

Supplementary MWAS were conducted on two additional depression-PRSs to investigate the associations found within the MHC region. Two additional PRSs were calculated using (1) the independent genetic risk variants reported in the depression GWAS by Howard et al. with a wider pruning window of 3 Mb and retaining only one variant in the MHC region and (2) SNPs located outside of MHC region, respectively. Analysis (1) was conducted to identify if the associations found in the MHC region were due to the additive effect of many genetic variants included in the MHC region. Analysis (2) was conducted to find out if the CpG associations found within the MHC region were conferred by genetic variants located *trans* to this region. The number of significant associations found within the MHC region for the PRS calculated using independent genetic risk variants reduced from 71 to 41, at the depression GWAS PRS *p*-value threshold of 5 × 10^−8^. No CpGs within the MHC region were found to be significantly associated with the PRS generated from variants mapping without the MHC region. See Additional file 1: Fig. S3.

Outside of the MHC region, 26 probes showed significant associations with depression-PRS estimated across the genome at pT of 5 × 10^−8^ (*p*_Bonferroni_ ranged from 0.049 to 2.41 × 10^−16^). The top ten probes are listed in Additional file 1: Table S5. Genes mapping near to the top probes were associated with histone deacetylase, DNA binding and transcriptional processes (such as *MAD1L1, TCF4* and *RERE*), and neuronal plasticity and growth (for example, *NEGR1*).

The effect sizes for the significant CpG probes showed high correlations between Set 1 and 2 (*r* = 0.84), and direction for all significant associations was consistent between sets. For these significant probes, the distance to the nearest depression risk locus was significantly lower than those that were not significant (significant versus not significant: standardised Cohen’s *d* = 0.920, *p* < 1 × 10^−32^). There were 12.7% of all significant CpGs located outside of 1 Mb boundaries of genetic risk loci for depression and outside of the region consisted of SNPs in LD (*R*^2^>0.1) with the genetic risk loci (see Additional file 2: Table S10).

### Replication depression-PRS MWAS in LBC1921, LBC1936 and ALSPAC

#### MWAS of depression-PRS on pT of 5 × 10^−8^ on adult and adolescent samples (LBC1921, LBC1936 and ALSPAC)

##### Replication in adults

**Fig. 3:**
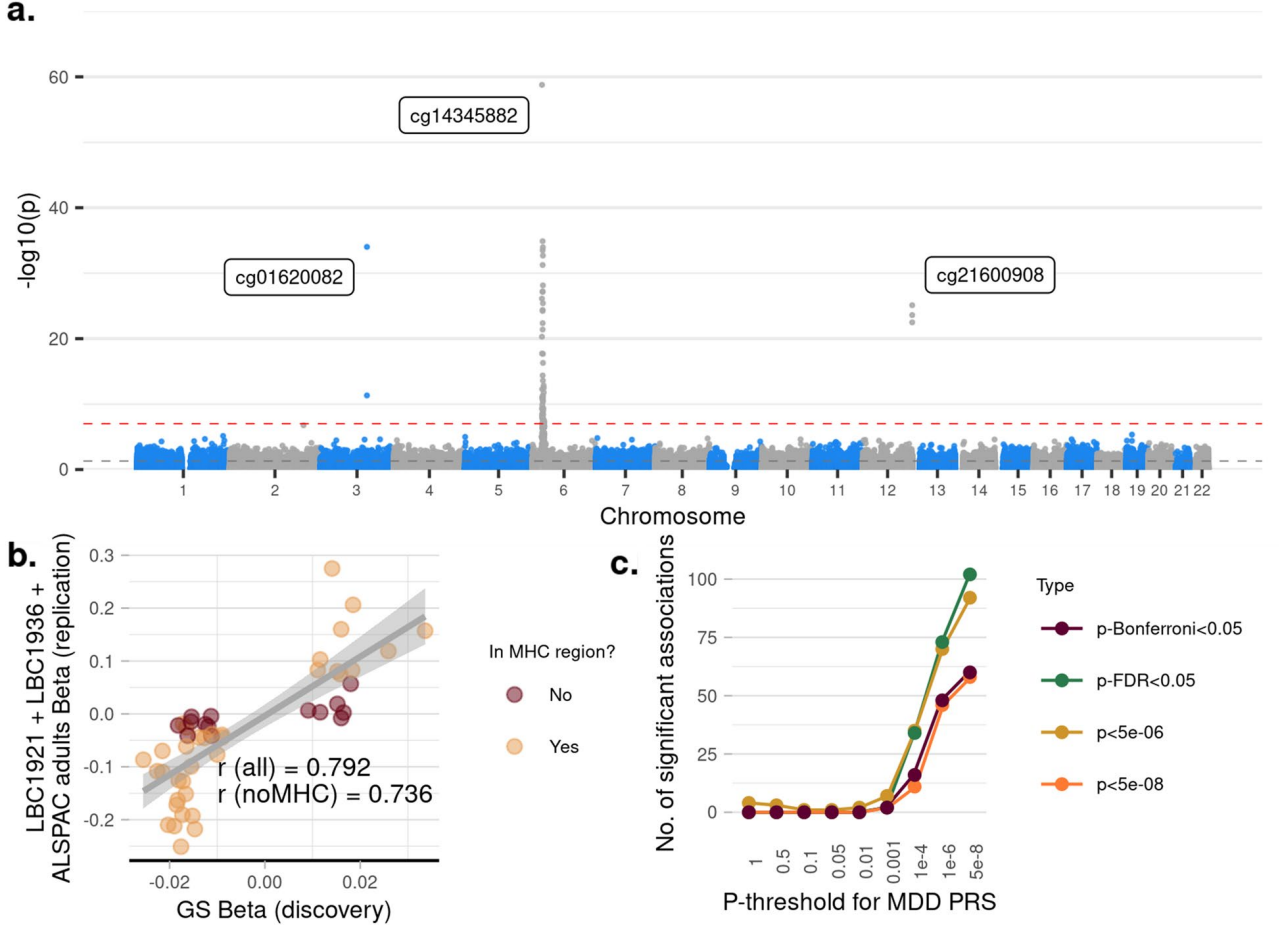
Replication MWAS in Lothian Birth Cohort (LBC) 1921, LBC1936 and Avon Longitudinal Study of Parents and Children (ALSPAC) adults. **a** Manhattan plot for the replication MWAS for PRS of pT at 5 × 10^−8^. Each dot represents a CpG probe. *X*-axis represents the relative position of the probes in the genome. *Y*-axis represents −log10-transformed *p*-values. The red dashed line represents the significance threshold for Bonferroni correction. **b** Scatter plot showing the correlation of standardised regression coefficients between the discovery (GS) and replication (LCB1921+LBC1936+ALSPAC adults) analysis. Each dot represents a CpG probe. Probes shown in the figure are those associated with depression-PRS of pT at 5 × 10^−8^ in the discovery MWAS (in GS). Dots in green represent probes locate in the major histocompatibility complex (*MHC*) regions and those in red represent other probes that locate outside of the MHC region **c** Number of CpG probes significantly associated with polygenic risk scores (PRS) at nine different pTs for replication analysis. *X*-axis represents the pTs used for generating PRS. *Y*-axis shows the number of probes significant associated with the given PRS. The four different lines represent four types of methods to define significance

We looked at a subset of CpG probes that were significant in the discovery MWAS analysis and found that the standardised effect sizes were correlated between the discovery and replication meta-MWAS of LBC1921, LBC1936 and ALSPAC adults, with (*N*_probe_ = 49, *r* = 0.79) or without the probes located in the MHC region (*N*_probe_ = 14, *r* = 0.74). There were 98.0% associations found in the discovery MWAS which remained in the same direction and 77.6% and 67.3% remained significant before and after Bonferroni correction within the replication analysis, respectively. See Fig. 3.

We then looked at the probes within and outside of the MHC region separately. Within the MHC region, effects for the top independent probe remained in the same direction and was significantly replicated (*p*_Bonferroni_ = 8.24 × 10^−58^). For the probes outside of the MHC region, 92.9% of the effects remained in the same direction, 35.7% were nominally significant and 7.1% were significant after Bonferroni correction.

##### Replication in adolescents

Standardised effect sizes for the significant CpG probes found in the discovery MWAS were highly correlated with those in the MWAS on adolescents from ALSPAC (all CpG probes: *N*_probe_ = 50, *r* = 0.81; no MHC region: *N*_probe_ = 14, *r* = 0.64. Effect for 89.8% of the probes remained in the same direction, 68.0% remained nominally significant and 46.0% were significant after Bonferroni correction.

Within the MHC region, effects for the top independent probe remained in the consistent direction and was significant (*p*_Bonferroni_ = 1.71 × 10^−12^). For the probes outside of the MHC region, effects for 85.7% of the probes remained in the same direction, 21.4% were nominally significant and 7.1% were significant after Bonferroni correction.

#### MWAS for depression-PRS on all p-thresholds on adult samples

Meta-analysis of the MWAS of depression-PRS for replication cohorts (LBC1921, LBC1936 and ALSPAC adults) showed that, for depression-PRS of pT at 5 × 10^−8^, 1 × 10^−6^, 1 × 10^−4^ and 0.001, the number of significant CpG probes were 60, 48, 16 and 2, respectively. Similar to the discovery analysis, no significant associations were found for PRS of pT ≥ 0.01.

A full list of results for replication analysis can be found in Additional file 1: Figs. S4-S7 and Additional file 2: Table S10.

### Pathway enrichment analysis

GO terms and KEGG pathways were assessed for the genes associated with depression-PRS of pT at 5 × 10^−8^. There were 118 enriched GO terms nominally significant but none was significant after FDR correction (*p*_min_ = 2.02 × 10^−3^). The majority of the nominally significant GO terms were associated with immune response and brain maturation. No enriched KEGG pathways reached significance (*p* > 0.089). The top ten GO terms and KEGG pathways are listed in Tables 1 and 2.

### SNP–CpG mapping for the depression risk loci

SNP-CpG probe associations were investigated by conducting MWAS for each of the independent genetic risk loci for depression. The analysis aimed to further inform individual associations between each genetic risk locus and DNA methylation. There were 3969 CpG probes that showed significant associations with at least one leading genetic risk variant after Bonferroni correction. Significant associations after Bonferroni correction are described below (*p* < 1.31 × 10^−7^).

**Fig. 4:**
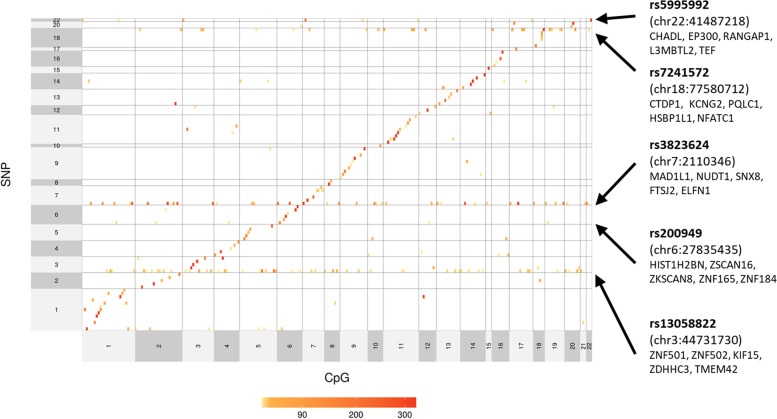
Heatmap showing the SNP-CpG mapping. Each row and column represents a CpG probe and depression risk locus, respectively. Those tests that are not significant after Bonferroni correction are left blank. For those significant associations, a darker cell represents a higher −log10-transformed *p*-value. All CpG probes and depression risk loci were categorised based on which chromosome (CHR) they locate. Within each chromosome, probes and SNPs are aligned from left to right or from bottom to top based on their genomic position

There were 94 of the 96 genetic risk variants tested showed significant *cis* association with CpGs within 1 Mb distance (see Fig. 4). There were 46 genetic risk variants (47.9% of all variants tested) that showed *trans* associations outside of their 1-Mb window and 33 variants (34.4% of all variants tested) that had *trans* associations with CpGs located on at least one different chromosome.

Five genetic loci showed associations with methylation levels at CpGs located in more than eight of the distal autosomal chromosomes (see Fig. 4). Genes close to these genetic risk variants were involved in, for example, nucleic acid transcription activities, which includes nucleic acid binding (*ZNF179* and *ESR2*), mitotic assembly (*MAD1L1*) and encoding proteins that colocalise with transcription factors (*RERE*). Regional association plots showing genes within 1 Mb distance from the five genetic variants can be found in Additional file 1: Fig. S8.

### Colocalisation analysis

We hypothesised that SNPs influencing risk for depression and those influencing DNAm would be shared. Colocalisation analysis, however, indicated that there was no strong evidence (PP_4_>0.8, PP_4_/PP_3_ > 5) [55] for shared genetic factors between loci for depression and DNAm. The posterior probability for one region was supportive of a suggestive colocalised association signal for both depression and DNAm in that region (PP_4_ = 0.71) [56]. Within this region, the SNP with the highest posterior probability of being shared SNPs for the two traits (66%) was rs73163796, which colocalised with genetic variation influencing a smoking-associated CpG site, cg15099418 [57]. Additional file 3 contains results for all 102 regions investigated in colocalisation analysis.

### MR

#### Discovery MR: causal effect of DNAm on depression using GoDMC data

Eight probes: cg06552810, cg07519229, cg14159747, cg14345882, cg14844989, cg19624444, cg23275840 and cg26647111, showed significant causal effect using the IVW and WM methods (absolute *β*_IVW_ ranged from 0.017 to 0.040, *p*_FDR_ ranged from 4.48 × 10^−3^to 3.44 × 10^−8^, absolute *β*_WM_ ranged from 0.012 to 0.038, *p*_FDR_ ranged from 1.75 × 10^−3^ to 2.07 × 10^−16^, *p*_FDR_ for Q-statistics ranged from 0.071 to 8.74 × 10^−7^). Effect sizes for the above probes were consistent between the IVW and WM methods. No significant causal effect on depression was found using the MR-Egger method for these probes (*β*_MR-Egger_ ranged from 0.004 to 0.034, *p*_FDR_ ranged from 0.703 to 0.319). However, the direction of effects remained the same with the IVW and WM methods and the test for MR-Egger intercept showed no evidence of horizontal pleiotropy (*p*_FDR_ for MR-Egger intercept ranged from 0.797 to 0.267).

**Fig. 5:**
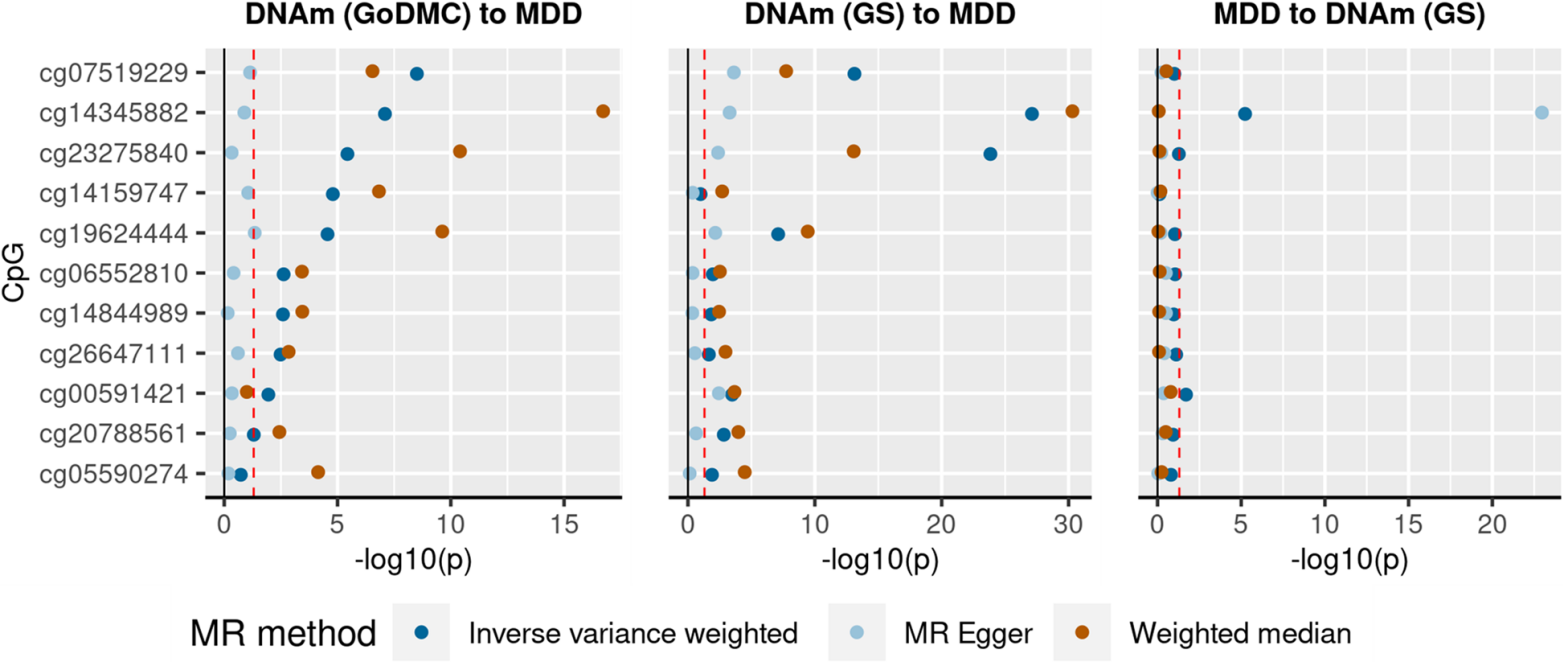
Mendelian randomisation (MR) analysis on DNAm and depression using data from the Genetics of DNA Methylation Consortium (GoDMC). **a** Discovery MR testing causal effect of DNAm to depression using GoDMC data. **b** Replication MR testing effect of DNAm using GS data to depression. **c** MR of reversed directionality testing the causal effect from depression to DNAm. *X*-axes represent *p*-values for MR analyses. *Y*-axes represent the individual tests conducted

Results are also shown in Fig. 5 and Additional file 1: Fig. S9 and Table S6.

#### Replication MR: causal effect of DNAm on depression using GS data

A replication MR was conducted to look at the causal effect of DNAm on depression, using an independent set of mQTL data. All of the potentially causal MR effects of DNAm to depression found in the discovery analysis showed consistent direction in the replication analysis and across all three MR methods. For all three MR methods, the effect sizes were highly correlated between discovery and replication analyses (*r* ranged from 0.641 to 0.953). Four out of eight significant effects found in the discovery MR analysis were significant for all three MR methods in the replication analyses (absolute *β*_IVW_ ranged from 0.048 to 0.199, *p*_IVW-FDR_ ranged from 1.20 × 10^−7^ to 1.15 × 10^−26^; absolute *β*_WM_ ranged from 0.032 to 0.192, *p*_WM-FDR_ ranged from 3.53 × 10^−3^ to 7.45 × 10^−30^). Three other probes (cg06552810, cg14844989 and cg26647111) showed significant causal effect at IVW and WM MR methods (see statistics in Additional file 1: Table S3). MR-Egger intercepts were not significantly deviated from 0 for all replication MR (*p*_FDR_ > 0.61), and thus showed no evidence of horizontal pleiotropy. See Additional file 1: Table S7.

#### Multi-variable MR: independent causal effect of DNAm on depression using GS data

We next tested for causal associations between DNAm at multiple CpGs from the discovery analysis to depression. The significant probes were entered into the two-sample MR analysis simultaneously, to identify the set of independent CpGs that showed the strongest and independent casual associations with depression using the IVW method. Three probes showed putatively causal effects when all CpGs were considered simultaneously. They are as follows: cg23275840 on chromosome 4, cg14345882 on chromosome 6 and cg14844989 on chromosome 11 (absolute β_IVW_ ranged from 0.129 to 3.028, *p*_FDR_ ranged from 0.025 to 2.21 × 10^−7^, see Additional file 1: Fig. S10 and Table S8). Genes annotated with these CpG probes are *BTN3A2* and *CORIN*. These genes are involved in signalling receptor binding in the brain and in hormonal regulation.

#### MR: causal effects of depression liability on DNA methylation

MR provided no consistent evidence of a causal effect of depression liability on DNA methylation. The effects to cg09256413 and cg16996682 were significant for the IVW method (absolute β_IVW_ ranged from 0.066 to 0.086, *p*_FDR_ ranged from 0.028 to 0.003), but the effects were not significant for neither WM nor MR-Egger methods (absolute *β*_WM/MR-Egger_ ranged from 0.003 to 0.251, *p*_FDR_>0.12). The effect from depression to cg14345882 was significant for both IVW and MR-Egger (*β*_IVW/MR-Egger_ − 0.004 to − 6.905, *p*_FDR_ < 8.85 × 10^−5^), but the effect was not significant for the WM method (*β*_WM_ = 0.004, *p*_FDR_ = 0.882) with an opposite direction to the other two methods, and there was strong evidence for heterogeneity between genetic instruments (*p* for Q-statistics <1 × 10^−328^). All other effects were not significant after FDR correction (*p*_FDR_ > 0.074).

See Fig. 5, Additional file 1: Fig. S11 and Table S9.

## Discussion

PRS for depression showed widespread associations with peripheral blood DNAm across the methylome in Generation Scotland: Scottish Family Health Study Cohort (GS, *N* = 8898). DNAm associations showed highly consistent results in the replication analysis in adults (*N* = 2049, *r*_*β*_ = 0.79) and in adolescents (*N* = 423, *r*_*β*_ = 0.81). Significant CpG probes are enriched in immunological processes in the human leukocyte antigen (HLA) system and neuronal maturation and development. Influence from the genetic risk of depression was both local (*cis*) and distal (*trans*). Five genetic risk loci showed widespread *trans* effect across more than eight of the autosomal chromosomes. Finally, using Mendelian randomisation (MR), we found evidence of a mutually causal effect of DNAm on liability to depression at CpG probes associated with PRS for depression.

The probes associated with genetic risk variants for depression map to genes including *TRIM27*, *BTN3A2* and *HIST1H2AI*. These HLA-related genes have been widely found associated with psychiatric conditions such as schizophrenia and bipolar disorder [6]. Other genes that located outside of the MHC region, such as *MAD1L1*, *RERE*, *SORCS3* and *ANKK1*, are associated with neuronal development and guidance of neuronal growth [58], transcriptional processes [59, 60] and other risk factors for depression, for example, obesity, smoking and abnormal physical development [61].

DNAm is associated with both genetic and environmental factors that collectively contribute to disease liability [62]. The present study focuses on investigating the associations between genetic risk of depression and DNAm, as well as the mechanism of genetic risk that penetrates through DNAm to disease liability. To our knowledge, this is the first such investigation using PRS for depression, which is likely to be due to a lack of statistical power within previously available samples. However, small-scale twin studies, although using a different design, have shown consistent findings with the present study. For example, differential methylation in CpG probes mapping near *MAD1L1* were found in affected depression patients compared with their unaffected monozygotic twins [63]. The finding was replicated later in a large-scale study of 724 twin pairs [64]. Compared to the previous and smaller-scale twin studies that showed small numbers of findings, the present study showed novel associations implicating genes involved in brain maturation and synaptic processing. This may indicate a broader mechanistic and potentially mediating role for DNAm in conferring the downstream effects of genetic risk. Our findings also highlight that DNAm may facilitate the functional interpretation of genetic risk loci.

MR provided evidence for a causal effect of methylation levels at CpG probes associated with lead SNPs on depression. After controlling for functional pleiotropy shared between CpG probes, three probes showed an independent causal effect on depression. Genes annotated with these independent probes are associated with phenotypes such as lower total brain volume [65], higher C-reactive protein [66], obesity [67] and adverse lifestyle factors such as smoking [68], which are implicated in both patients with depression and those who have been exposed to early environmental risks, such as childhood trauma [69]. These phenotypes have also been shown to have causal effects on depression in previous MR studies [4, 5].

Statistical evidence was stronger for the causal effect from DNAm to depression compared to the opposite direction, despite that more genetic variants were used for the reversed causal effect, and thus statistical power was greater (*N*_SNP_ for DNAm to depression ranged from 4 to 22 and *N*_SNP_ for depression to DNAm was 122). The highly consistent methylome-wide associations found across adults and adolescents may indicate that early genetic influence on DNAm result in a predominantly directional effect from DNAm to depression [70].

The present study utilises large samples with replication analyses yielding highly consistent results. One limitation for interpreting the current findings is that DNAm data was collected from blood samples that may not reflect the most relevant cell types in depression. Nevertheless, studies have shown that the genetic drivers of DNAm have similar effects across multiple cell types [13, 71]. The greater accessibility of DNAm from whole blood also has clear sample size and other methodological advantages compared to measures obtained from neural tissue post-mortem, and it is more likely that these measures could be used in future clinical applications. Future studies could further expand the scope by including other cell and tissue types. In addition, findings from the present study were supported by European samples. Future studies regarding other ancestry groups are necessary for identifying more generalisable genetic-epigenetic associations.

## Conclusions

In the current study, we demonstrate that genome-wide genetic risk variants for depression show widespread methylome-wide DNAm associations both individually and when combined in a risk score. These changes implicate antigen processing and immune system responses and may provide clues to the underlying mechanisms of depression.

## Supplementary Information

**Additional file 1: Figure S1.** Manhattan plots for discovery MWAS on GS. **Figure S2.** Quantile-quantile plots for methylome-wide association studies (MWAS) of polygenic risk scores (PRS) for depression at p threshold of 5e-8 on Generation Scotland: Scottish Family Health Study (GS). **Figure S3.** Supplementary MWAS investigating the MHC region. **Figure S4.** Manhattan plots for replication MWAS on LBC 1921, LBC 1963 and ALSPAC adults. **Figure S5.** Quantile-quantile plots for replication MWAS of PRS for depression at p threshold of 5e-8 on Lothian Birth Cohort (LBC) 1921, LBC 1936 and Avon Longitudinal Study of Parents and Children (ALSPAC) adults. **Figure S6.** Manhattan plots for replication MWAS ALSPAC children. **Figure S7.** Quantile-quantile plots for replication MWAS of PRS for depression at p threshold of 5×10-8 on ALSPAC children. **Figure S8.** Regional association plots for the genetic loci showed associations with methylation levels at CpGs located in more than half of the distal autosomal chromosomes (window = 1Mb). **Figure S9.** Scatter plot for discovery Mendelian randomisation (MR) of DNA methylation (DNAm) to depression. **Figure S10.** Scatter plot for replication MR of DNAm to depression. **Figure S11.** Scatter plot for MR of liability of depression to DNAm. **Figure S12.** Supplementary MWAS on the unrelated sample in GS (N=6777). **Table S1.** Number of genetic variants used for calculating PRSs. **Table S2.** Association between PRSs and prevalence depression. **Table S3.** Association between PRSs and DNAm-estimated white-blood cell proportions. **Table S4.** Genomic inflation factor for discovery, adult replication (LBC 1921 + LBC 1936 + ALSPAC adults) and adolescent replication (ALSPAC adolescents) MWAS. **Table S5.** Top CpG probes associated with PRS at pT = 5e-8. **Table S6.** Results for discovery MR of DNAm to depression. **Table S7.** Results for replication MR of DNAm to depression. **Table S8.** Results for multivariable MR of DNAm to depression. **Table S9.** Results for MR of liability of depression to DNAm.

**Additional file 2: Table S10.** Association statistics for the discovery MWAS and replication MWASs in the adult and adolescent samples.

**Additional file 3.** Results for colocalisation analysis between depression and mQTL.

## Data Availability

All codes used for generating the PRS, preparing genetic data and analysis have been stored in a publicly available GitHub repository in the GitHub repository: https://github.com/xshen796/MDD_PRS_MWAS [53]. A detailed summary of scripts used for each analysis can be found in the wiki page for the GitHub repository: https://github.com/xshen796/MDD_PRS_MWAS/wiki. Summary statistics for the association analyses conducted in the present study can be found in Additional file 2: Table S10. Summary statistics for depression GWAS that was used for generating the PRS can be found in the URL: https://datashare.ed.ac.uk/handle/10283/3203. PRS for depression has been previously developed and validated by Howard et al. [33] in GS. According to the terms of consent, access to any form of individual-level data requires application for each individual cohort. Access to individual-level genetic, DNAm data and phenotypes need to be approved by the GS Access Committee (https://www.ed.ac.uk/generation-scotland/for-researchers/access, mailto: access@generationscotland.org). Data dictionary for GS is available at the URL: https://datashare.ed.ac.uk/handle/10283/2988. Access to LBC1921 and LBC1936 must approved by the LBC research team. A guideline for accessing LBC data can be found in the URL: https://www.ed.ac.uk/lothian-birth-cohorts/data-access-collaboration. Data structure, application procedure and contact details are described in the guideline. Access to ALSPAC data requires approved application. Data dictionary and requirements for data access are described in detail in the URL: http://www.bristol.ac.uk/alspac/researchers/access/.

## Abbreviations

DNAm: DNA methylation
MWAS: Methylome-wide association study
CpG: Cytosine-guanine dinucleotide
PRS: Polygenic risk score(s)
MR: Mendelian randomisation
GS: Generation Scotland: Scottish Family Health Study
LBC: Lothian Birth Cohort
ALSPAC: Avon Longitudinal Study of Parents and Children
GoDMC: Genetics of DNA Methylation Consortium
SD: Standard deviation
mQTL: Methylation quantitative trait loci
MHC: Major histocompatibility complex
HLA: Human leukocyte antigen
