# Supplementary material for: DNA methylome-wide association study of genetic risk for depression implicates antigen processing and immune responses
Source: Genome Med. 2022 Mar 31;14:36. doi: 10.1186/s13073-022-01039-5 (PMC8969265; doi:10.1186/s13073-022-01039-5)
Supplement: Supplementary file 1 — Additional file 1: Figure S1. Manhattan plots for discovery MWAS on GS. Figure S2. Quantile-quantile plots for methylome-wide association studies (MWAS) of polygenic risk scores (PRS) for depression at p threshold of 5e-8 on Generation Scotland: Scottish Family Health Study (GS). Figure S3. Supplementary MWAS investigating the MHC region. Figure S4. Manhattan plots for replication MWAS on LBC 1921, LBC 1963 and ALSPAC adults. Figure S5. Quantile-quantile plots for replication MWAS of PRS for depression at p threshold of 5e-8 on Lothian Birth Cohort (LBC) 1921, LBC 1936 and Avon Longitudinal Study of Parents and Children (ALSPAC) adults. Figure S6. Manhattan plots for replication MWAS ALSPAC children. Figure S7. Quantile-quantile plots for replication MWAS of PRS for depression at p threshold of 5×10^-8^ on ALSPAC children. Figure S8. Regional association plots for the genetic loci showed associations with methylation levels at CpGs located in more than half of the distal autosomal chromosomes (window = 1Mb). Figure S9. Scatter plot for discovery Mendelian randomisation (MR) of DNA methylation (DNAm) to depression. Figure S10. Scatter plot for replication MR of DNAm to depression. Figure S11. Scatter plot for MR of liability of depression to DNAm. Figure S12. Supplementary MWAS on the unrelated sample in GS (N=6777). Table S1. Number of genetic variants used for calculating PRSs. Table S2. Association between PRSs and prevalence depression. Table S3. Association between PRSs and DNAm-estimated white-blood cell proportions. Table S4. Genomic inflation factor for discovery, adult replication (LBC 1921 + LBC 1936 + ALSPAC adults) and adolescent replication (ALSPAC adolescents) MWAS. Table S5. Top CpG probes associated with PRS at pT = 5e-8. Table S6. Results for discovery MR of DNAm to depression. Table S7. Results for replication MR of DNAm to depression. Table S8. Results for multivariable MR of DNAm to depression. Table S9. Results for MR of liability of depression to [file 13073_2022_1039_MOESM1_ESM.docx]

Additional File 1

Methylome-wide association analysis of polygenic risk scores for depression

Shen X. et al.

## Supplementary Methods

### Additional information for Avon Longitudinal Study of Parents and Children (ALSPAC)

Pregnant women resident in Avon, UK with expected dates of delivery 1st April 1991 to 31st December 1992 were invited to take part in the study. The initial number of pregnancies enrolled is 14,541 (for these at least one questionnaire has been returned or a “Children in Focus” clinic had been attended by 19/07/99). Of these initial pregnancies, there was a total of 14,676 foetuses, resulting in 14,062 live births and 13,988 children who were alive at 1 year of age. When the oldest children were approximately 7 years of age, an attempt was made to bolster the initial sample with eligible cases who had failed to join the study originally. As a result, when considering variables collected from the age of seven onwards (and potentially abstracted from obstetric notes) there are data available for more than the 14,541 pregnancies mentioned above. The number of new pregnancies not in the initial sample (known as Phase I enrolment) that are currently represented on the built files and reflecting enrolment status at the age of 24 is 913 (456, 262 and 195 recruited during Phases II, III and IV respectively), resulting in an additional 913 children being enrolled. The phases of enrolment are described in more detail in the cohort profile paper and its update (see footnote 4 below). The total sample size for analyses using any data collected after the age of seven is therefore 15,454 pregnancies, resulting in 15,589 foetuses. Of these 14,901 were alive at 1 year of age. A 10% sample of the ALSPAC cohort, known as the Children in Focus (CiF) group, attended clinics at the University of Bristol at various time intervals between 4 to 61 months of age. The CiF group were chosen at random from the last 6 months of ALSPAC births (1432 families attended at least one clinic). Excluded were those mothers who had moved out of the area or were lost to follow-up, and those partaking in another study of infant development in Avon.

Study data were collected and managed using REDCap electronic data capture tools hosted at the University of Bristol^1^. REDCap (Research Electronic Data Capture) is a secure, web-based software platform designed to support data capture for research studies.

### Covarying confounders in the methylome-wide association study (MWAS)

#### Generation Scotland: **Scottish Family Health Study (GS)**

Technical confounders were pre-corrected. This was achived by residualising m-values against genetic relationship matrix (GRM), batch and estimated cell proportions. GRM was generated using PLINK2.0^2^ and was corrected in set 1 only, as participants were unrelated in set 2. Other covariates were fitted in the MWAS regression model, which include: age, sex, pack years, ever smoked tobacco and the first 20 principal components of the methylation data.

Ever smoked tobacco was an ordinal variable. Participants were asked to choose from one of the following responses: ‘Yes, currently smoke’ (= 3), ‘Yes, but stopped within the past 12 months’ (= 2), ‘Yes, but stopped for more than 12 months ago’ (= 1) and ‘No, never smoked’ (= 0). This variable was set as factor in all analyses using R.

Principal components of the methylation data was derived using the ‘FactoMineR’ R package (version 2.4)^3^. This step was conducted on the m-values pre-corrected for technical confounders.

#### Lothian Birth Cohort (LBC) 1921 and LBC 1936

All participants were unrelated within each cohort itself and between the two cohorts. All covariates were fitted in the MWAS regression model, which include: estimated cell proportions, batch, age, sex, ever smoked tobacco and the first 20 principal components of the methylation data.

Ever smoked tobacco variable was ordinal and thus was set as factor in all analyses. Responses include: ‘Current smoker’ (= 2), ‘Previous smoker’ (= 1) and ‘Never smoked’ (= 0).

Principal components of the methylation data was derived using the same method as GS.

#### ALSPAC

MWAS was conducted on parents and youth respectively. For each of the MWAS, only unrelated participants were included in the analyses. Procedures for selecting unrelated sample can be found in^4^. Covariates that were fitted in the MWAS analysis include: estimated cell proportions, age, sex, ever smoked tobacco, the first 15 variables derived from surrogate variable analysis and the first 10 genetic principal components.

Ever smoked tobacco was asked around the time of blood draw for DNAm processing. Participants were asked to choose from either smoked (= 1) or not smoked (= 0).

### Meta-analysis on MWAS results

Meta-analysis was conducted using METAL (version published on 25/03/2011)^5^ using the fixed-effect inverse-variance method.

For each individual analyses in the discovery and replication MWAS, meta-analysis was conducted to obtain an overall summary statistics of MWAS results. In the discovery analysis, MWAS on set 1 and set 2 GS data was conducted separately and then meta-analysed. In the replication analysis, MWAS was conducted on the two LBC cohorts together and ALSPAC adults. Summary statistics for replication analyses were then meta-analysed.

A final meta-analysis on the discovery and replication MWAS (LBC cohorts and ALSPAC adults) was conducted and presented in the Supplementary Information.

As there was extensive control for relatedness and population structure for MWAS in all cohorts, genomic control correction was not included in the analyses.

### Supplementary MWAS on the unrelated participants in GS

Relatedness in GS was accounted for using the m-values residualised against GCTA-estimated genomic relationship matrix in a mixed linear model. This approach maximises statistical power by retaining related participants in the analysis while controlling for relatedness. In addition to this approach, we conducted a supplementary MWAS on unrelated participants. For Set 1, there were 2636 unrelated participants retained (kinship coefficient < 0.05). Set 2 were unrelated (kinship coefficient < 0.05, N = 4141) within the set and with all participants in Set 1. Set 1 and 2 were then meta-analysed as described in the Methods. In total, 6777 unrelated participants were included in the analysis. In this supplementary MWAS, all CpG probes that were found associated with depression-PRS of p-value threshold (pT) at 5×10_-8_ in the main analysis remained nominally significant, and 47.9% of the probes remained significant after Bonferroni-correction across the entire methylome. All the effect sizes were in the same direction. Effect sizes for the analysis on the full sample and unrelated sample were highly correlated (r = 0.996 for all CpG probes and r = 0.997 for the probes outside of the MHC region). See Figure S12.

### Statistics for methylation quantitative trait loci (mQTL)

#### **Genetics of DNA Methylation Consortium** (GoDMC)

GoDMC(<http://www.godmc.org.uk/cohorts.html>) was established with the view of bringing together researchers with an interest in studying the genetic basis of DNA methylation variation, to consolidate as many resources and expertise as possible and thereby expedite this field of research^6^[.](https://www.medrxiv.org/content/10.1101/2020.09.01.20180406v1%7D.) The initial release of their findings consists of mQTL associations based on a sample size of 27,750 individuals. For the present study, LBC 1921, LBC 1936, GSK, and Brisbane Systems Genetics Study were removed from the mQTL meta-analysis as they were also included in the depression GWAS by Howard *et al.*^7^. Details for the cohorts can be found at the GoDMC website . As a result, a total of ~25,000 participants were left in the meta-analysis, with no overlapping participants with GS and no overlapping cohort with the GWAS for depression^7^.

*Genotype data*

Genotype data of all autosomes and chromosome X (if available) was imputed to 1000G reference panel and above using hg19/build37. Genotype data was filtered on an info score of 0.8 and a minor allele frequency (MAF) of 0.01. Genotype data was converted to best-guess data without a probability cut-off.

*DNA methylation data*

DNA methylation was measured in whole blood or cord blood using Illumina 450K or EPIC Beadchips in at least 100 European individuals. Normalized beta values were used, preferable normalized with the R package ‘meffil’ (version 1.1.1)8. Most analysts used ‘meffil’ to quality control and normalize the DNA methylation data using functional normalization. Protocols can be found here: <https://github.com/perishky/meffil/wiki>.

A github pipeline was implemented to run the analyses locally (<https://github.com/MRCIEU/godmc>) For the genotype data, several standard sample QC steps were performed including a sex check, removal of samples with >5% missingness, and the identification and exclusion of ancestry outliers. In datasets of ostensibly unrelated individuals, those that were found to be related (identity by state >0.125) were excluded.

The pipeline then residualised the normalized methylation betas by replacing outliers that were 10 standard deviations from the mean (3 iterations) with the probe mean, rank transforming the normalized beta values and regressing out age, sex, predicted cell counts, predicted smoking, genetic principal components and non-genetic methylation principal components. In family-based cohorts, genetic relatedness matrices were constructed and relatedness adjusted for using the ‘GRAMMAR’ approach. Genomic lambdas were checked by performing a GWAS of probe cg07959070. These residualised methylation measurements were used in all analyses.

*Association analysis*

First, every study performed a full analysis of all candidate mQTL associations, returning only associations at a threshold of p<1×10^-5^. All candidate mQTL associations at p<1×10_-5_ were combined to create a unique ‘candidate list’ of mQTL associations. In total, 102,965,711 candidate mQTL associations in *cis* (p<1×10^-5^, SNP located within 1Mb of the methylation site) and 710,638,230 candidate mQTL associations in *trans* were identified in at least one dataset. To avoid computational burden, we included *cis* associations found in at least one dataset and *trans* associations in at least two datasets. The candidate list (n=120,212,413) was then sent back to all cohorts and the association estimates obtained for every mQTL association on the candidate list. Meta analyses were run using a modified version of METAL using fixed-effect inverse-variance method^5^.

#### GS

Analysis of mQTL was conducted on DNAm data in GS for set 1 and set 2 separately. The OmicS-data-based Complex trait Analysis package was used for deriving mQTL summary statistics (<https://cnsgenomics.com/software/osca/>)^9^. DNAm data and covariates were kept consistent with the MWAS. Genetic data used for the mQTL analysis was also used for calculating polygenic risk scores for depression. Finally, meta-analysis between set 1 and set 2 was conducted on the mQTL summary statistics for each CpG probe.

## References

1. Harris, P. *et al.* Research electronic data capture (redcap)A metadata-driven methodology and workflow process for providing translational research informatics support. *Journal of Biomedical Informatics* **42**, 377–381 (2009).

2. Yang, J., Lee, S., Goddard, M. & Visscher, P. GCTA: A tool for genome-wide complex trait analysis. *The American Journal of Human Genetics* **88**, 76–82 (2011).

3. Lê, S., Josse, J. & Husson, F. FactoMineR: AnRPackage for multivariate analysis. *Journal of Statistical Software* **25**, (2008).

4. Caramaschi, D. *et al.* Epigenome-wide association study of seizures in childhood and adolescence. *Clinical Epigenetics* **12**, (2020).

5. Willer, C. J., Li, Y. & Abecasis, G. R. METAL: Fast and efficient meta-analysis of genomewide association scans. *Bioinformatics* **26**, 2190–2191 (2010).

6. Min, J. *et al.* Genomic and phenomic insights from an atlas of genetic effects on dna methylation. (2020).

7. Howard, D. *et al.* Genome-wide meta-analysis of depression identifies 102 independent variants and highlights the importance of the prefrontal brain regions. *Nature Neuroscience* **22**, 343–352 (2019).

8. Min, J., Hemani, G., Davey Smith, G., Relton, C. & Suderman, M. Meffil: Efficient normalization and analysis of very large dna methylation datasets. *Bioinformatics* (2018) doi:[10.1093/bioinformatics/bty476](https://doi.org/10.1093/bioinformatics/bty476).

9. Zhang, F. *et al.* OSCA: A tool for omic-data-based complex trait analysis. *Genome Biology* **20**, (2019).

#####


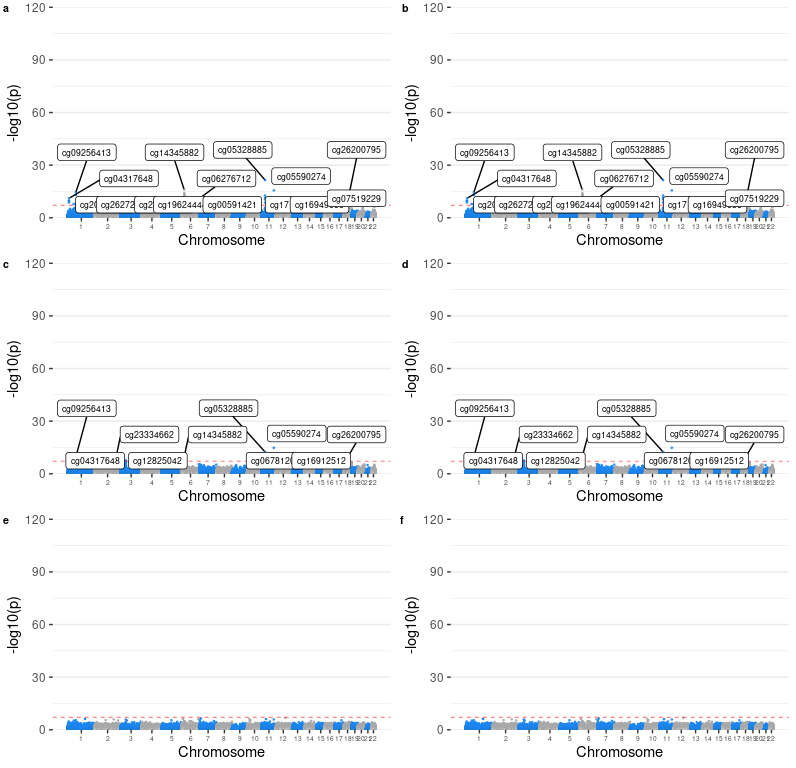


Figure S1 (a-f). Manhattan plots for discovery MWAS on GS. Panels (a-i) are for PRSs at p thresholds 5×10_-8_, 1×10_-6_, 1×10_-4_, 1×10_-3_, 0.005 and 0.01.


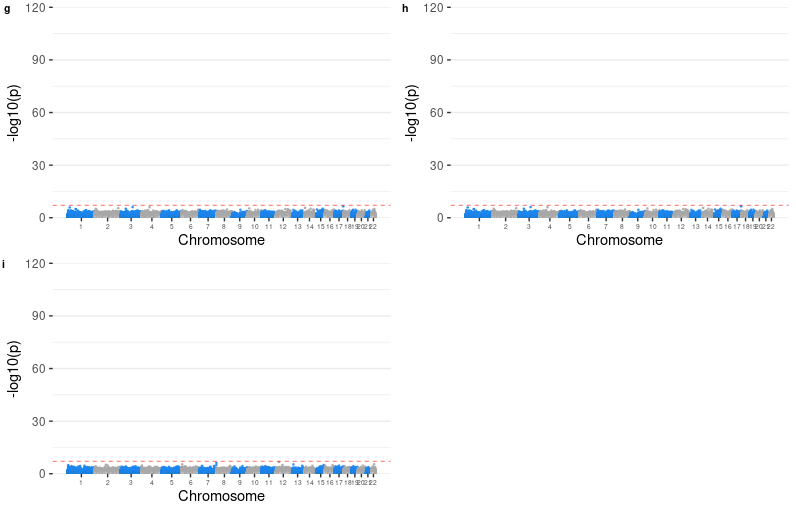


Figure S1 (g-i). Manhattan plots for discovery MWAS on GS. Panels (a-i) are for PRSs at p thresholds 0.05, 0.1, 0.5 and 1.

#####


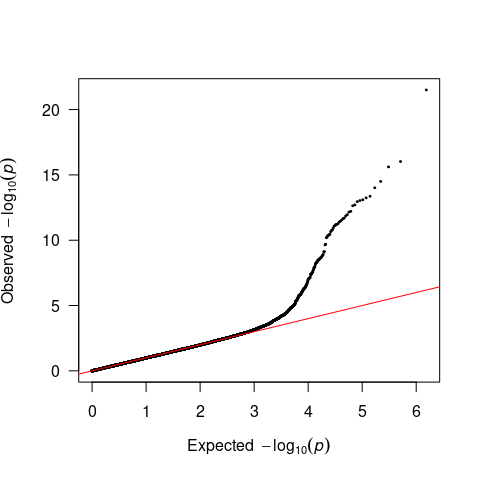


Figure S2. Quantile-quantile plots for methylome-wide association studies (MWAS) of polygenic risk scores (PRS) for depression at p threshold of 5×10_-8_ on Generation Scotland: Scottish Family Health Study (GS).

#####


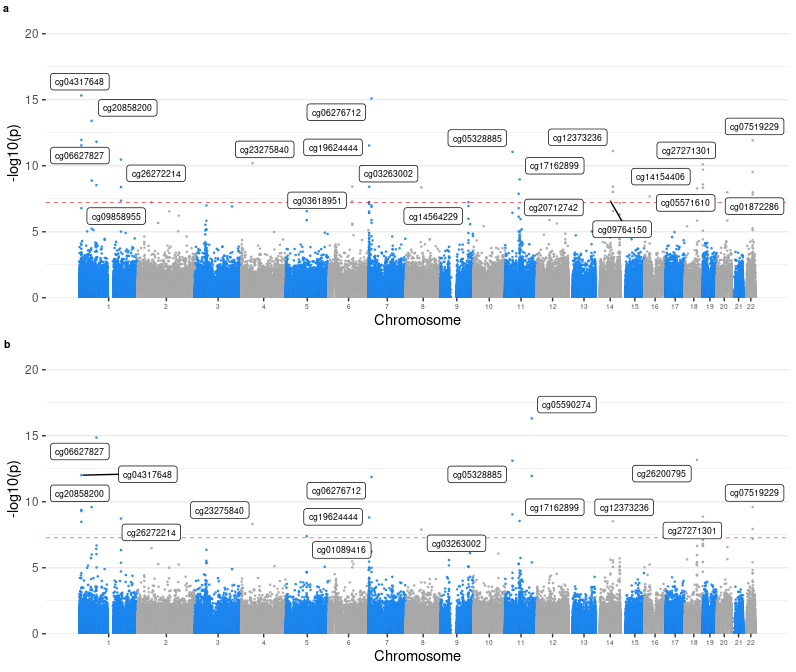


Figure S3. Supplementary MWAS investigating the MHC region. (a) MWAS for PRS calculated using the leading genetic risk variants. (b) MWAS for PRS calculated using genome-wide significant variants located outside of the MHC region.

#####


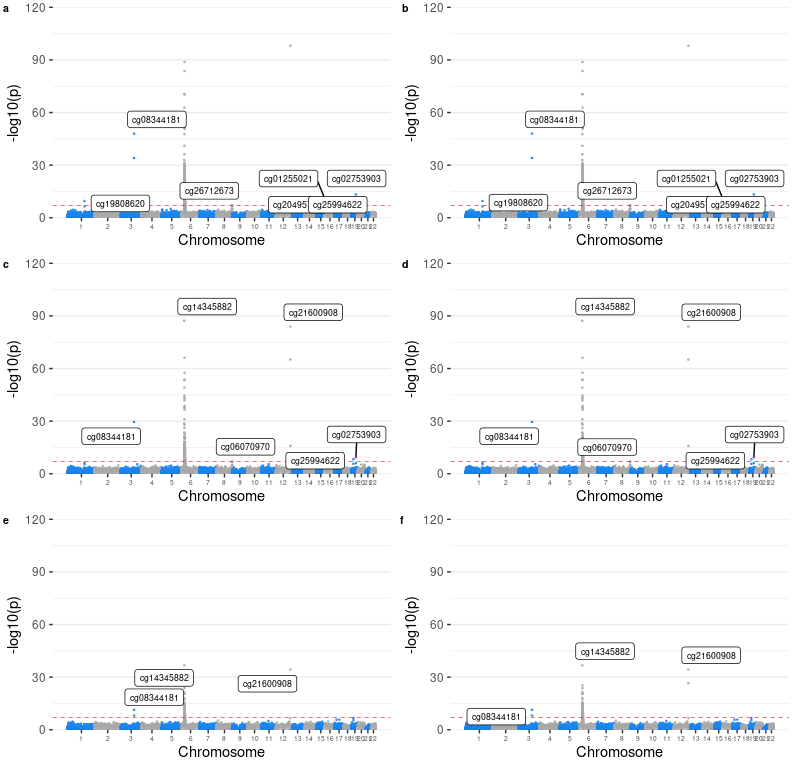


Figure S4 (a-f). Manhattan plots for replication MWAS on LBC 1921, LBC 1963 and ALSPAC adults. Panels (a-i) are for PRSs at p thresholds 5×10_-8_, 1×10_-6_, 1×10_-4_, 1×10_-3_, 0.005 and 0.01.

#####


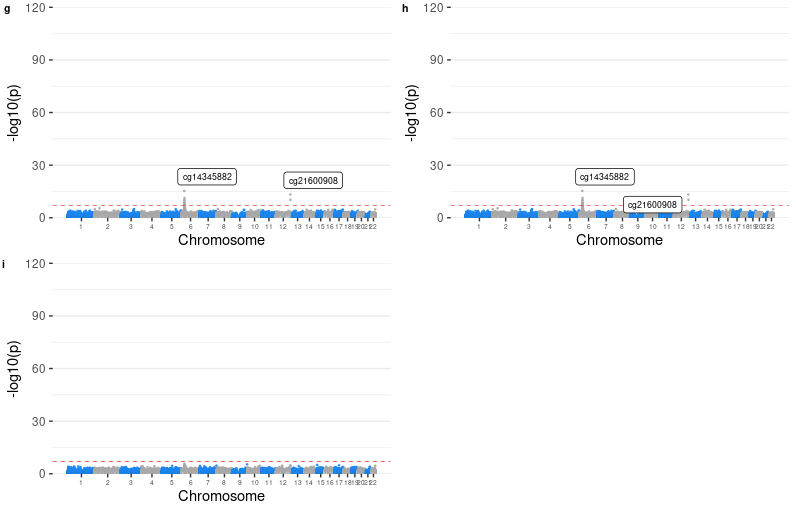


Figure S4 (g-j). Manhattan plots for replication MWAS on LBC 1921, LBC 1963 and ALSPAC adults. Panels (a-i) are for PRSs at p thresholds 0.05, 0.1, 0.5 and 1.

#####


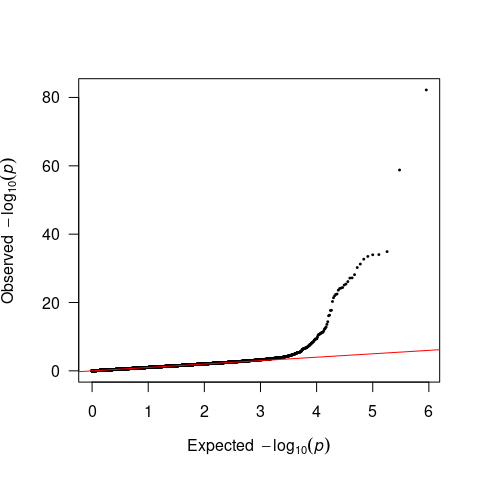


Figure S5. Quantile-quantile plots for replication MWAS of PRS for depression at p threshold of 5×10_-8_ on Lothian Birth Cohort (LBC) 1921, LBC 1936 and Avon Longitudinal Study of Parents and Children (ALSPAC) adults.

#####


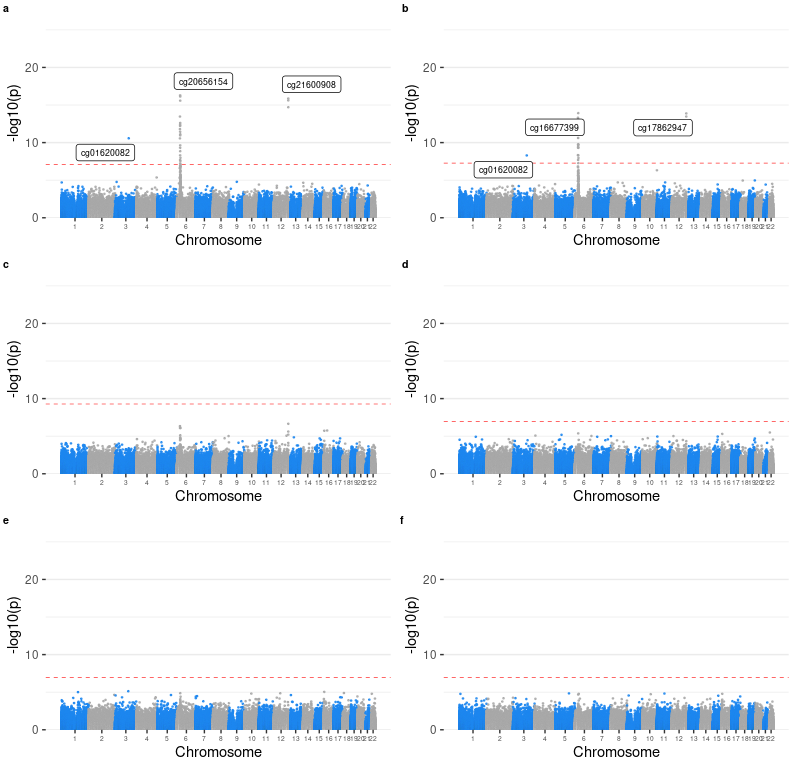


Figure S6 (a-f). Manhattan plots for replication MWAS ALSPAC children. Panels (a-i) are for PRSs at p thresholds 5×10_-8_, 1×10_-6_, 1×10_-4_, 1×10_-3_, 0.005 and 0.01.

#####


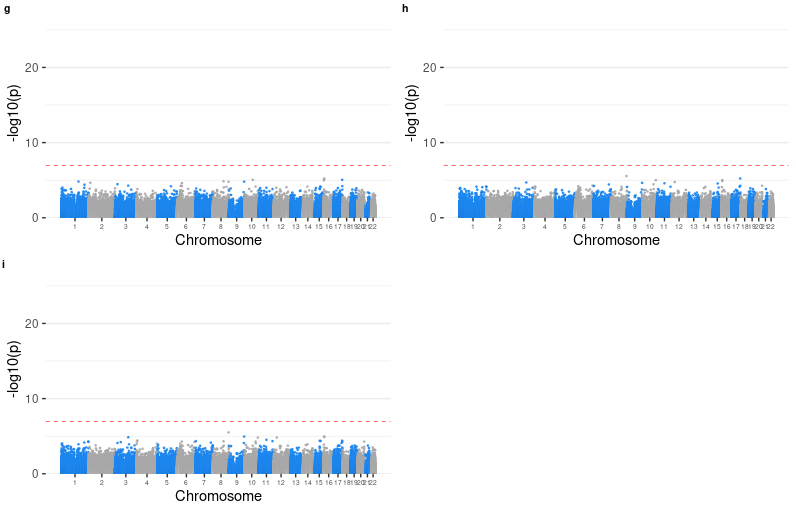


Figure S6 (g-j). Manhattan plots for replication MWAS ALSPAC children. Panels (a-i) are for PRSs at p thresholds 0.05, 0.1, 0.5 and 1.

#####


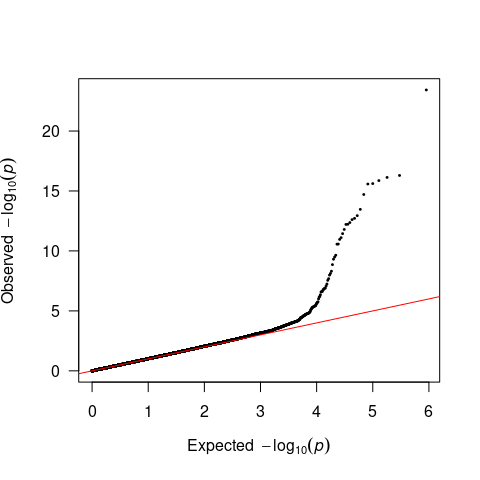


Figure S7. Quantile-quantile plots for replication MWAS of PRS for depression at p threshold of 5×10_-8_ on ALSPAC children.

#####


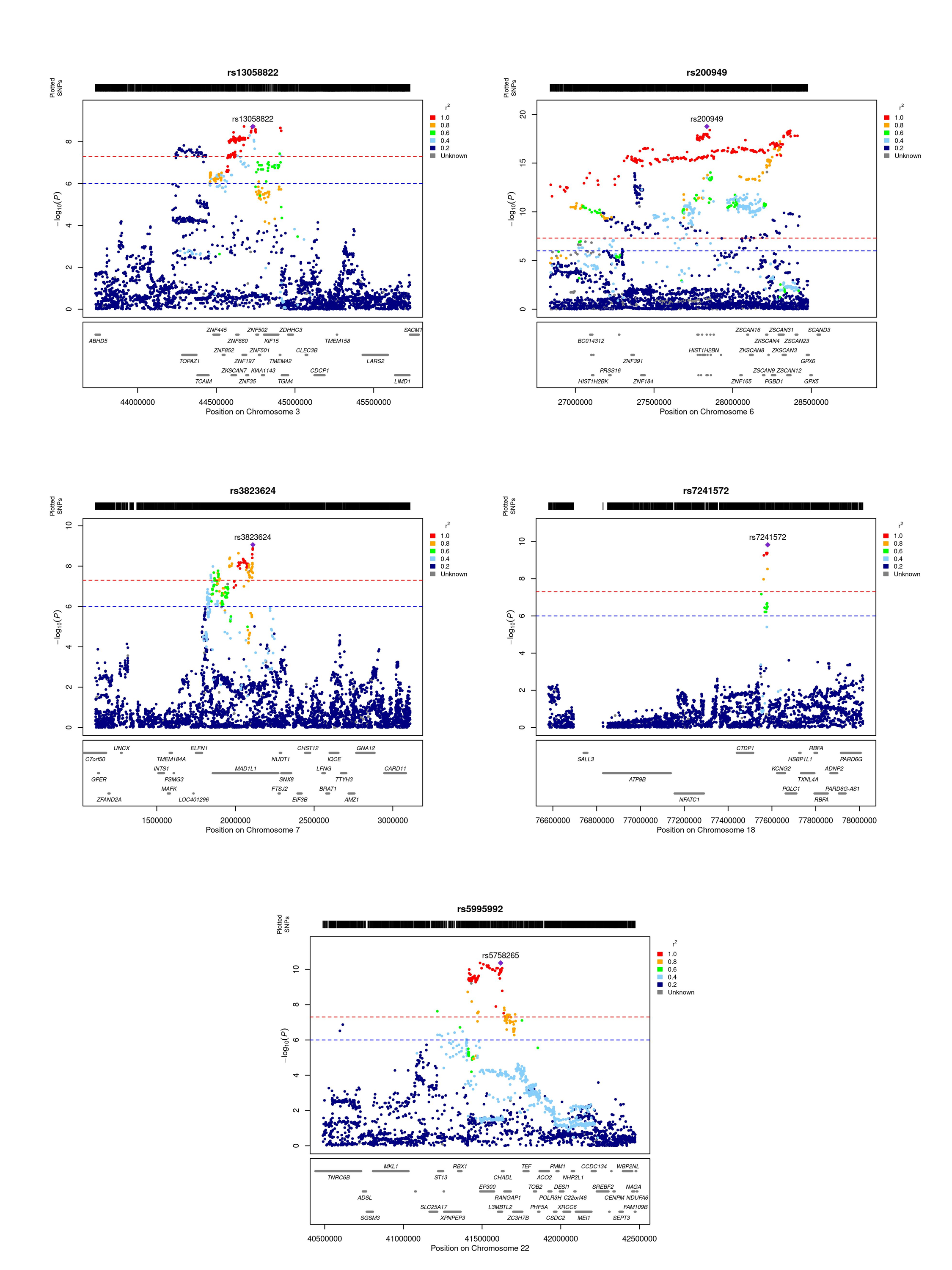


Figure S8. Regional association plots for the genetic loci showed associations with methylation levels at CpGs located in more than half of the distal autosomal chromosomes (window = 1Mb).

#####


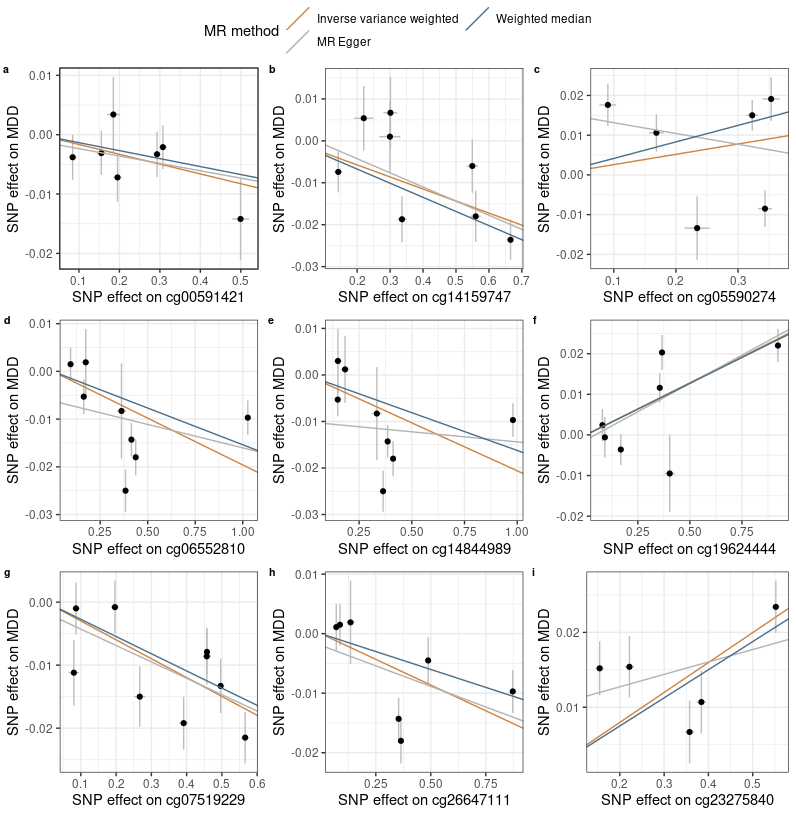


Figure S9. Scatter plot for discovery Mendelian randomisation (MR) of DNA methylation (DNAm) to depression (a-i).

#####


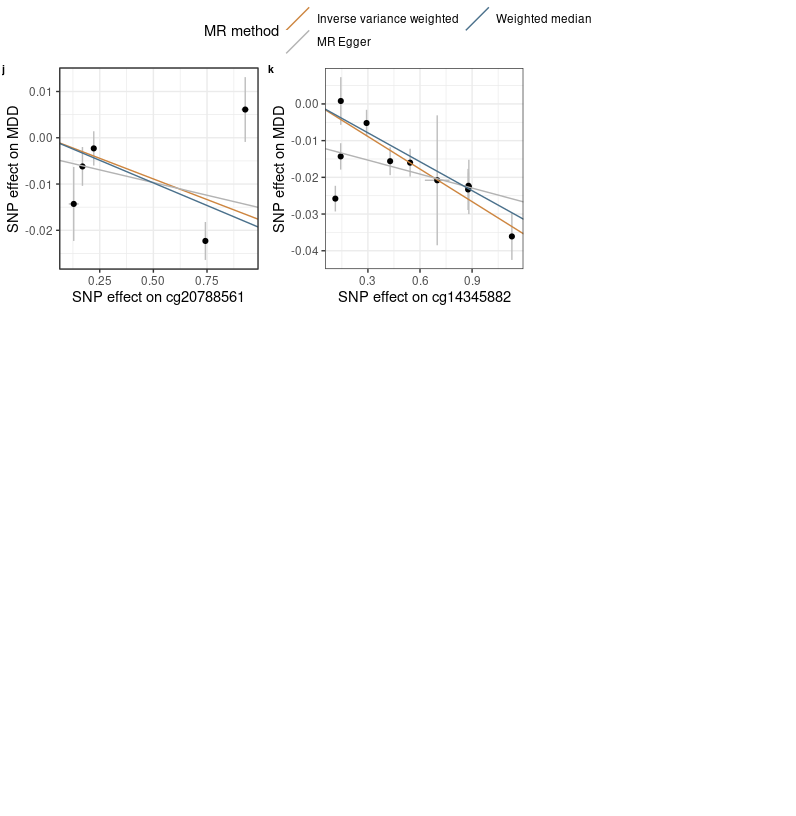


Figure S9. Scatter plot for discovery Mendelian randomisation (MR) of DNA methylation (DNAm) to depression (j-k).

#####


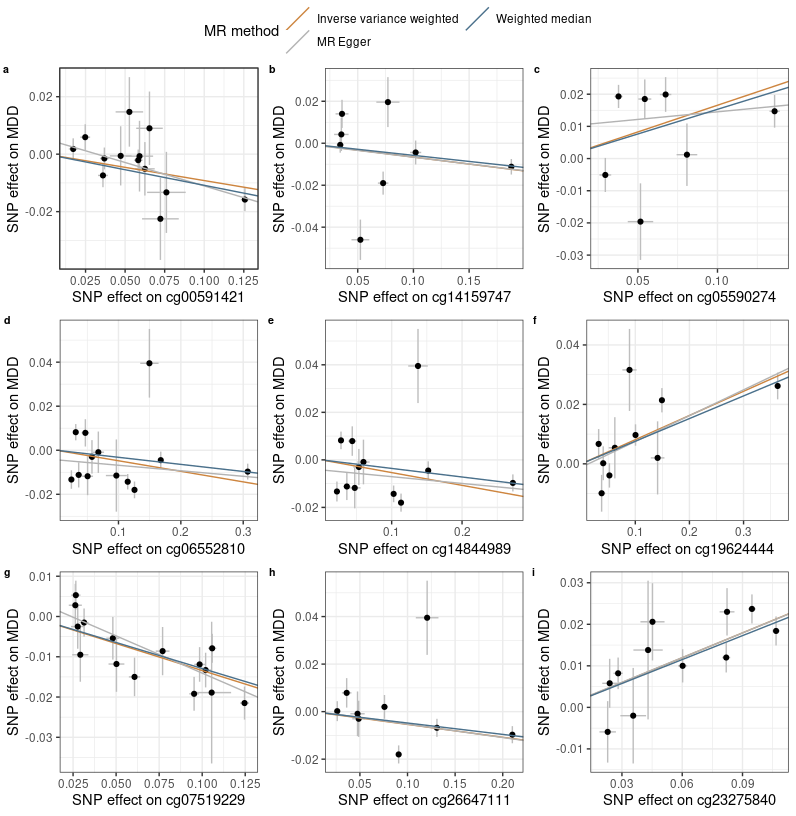


Figure S10. Scatter plot for replication MR of DNAm to depression (a-i).

#####


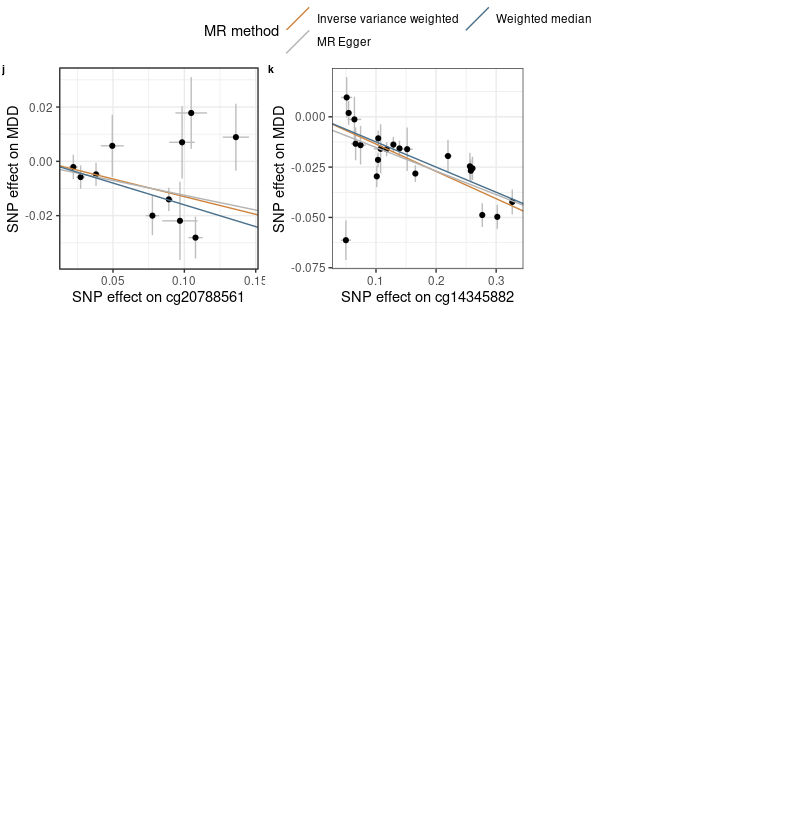


Figure S10. Scatter plot for replication MR of DNAm to depression (j-k).

#####


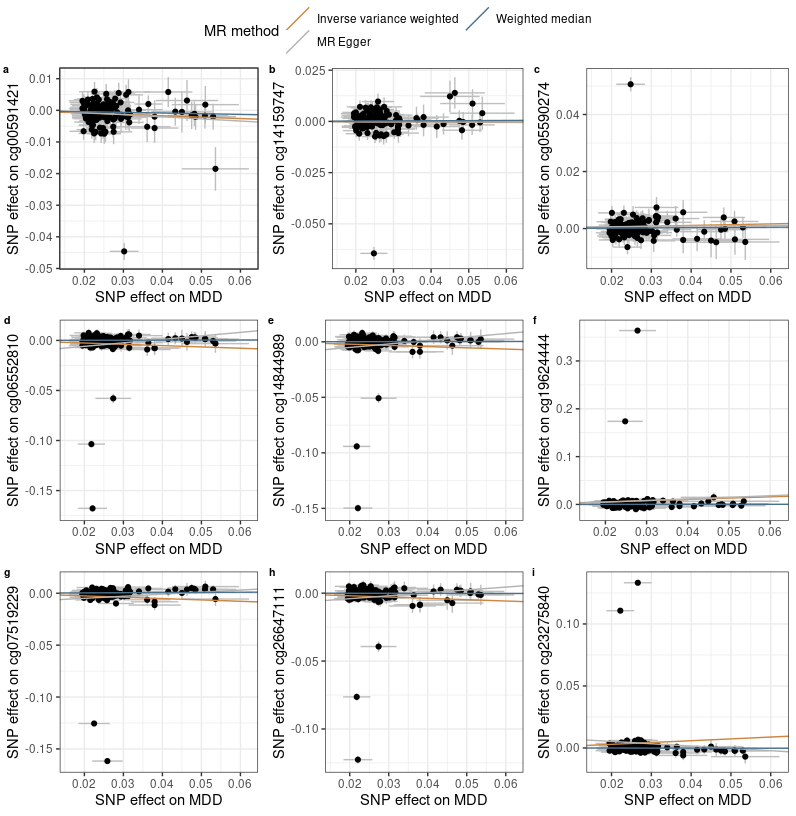


Figure S11. Scatter plot for MR of liability of depression to DNAm (a-i).

#####


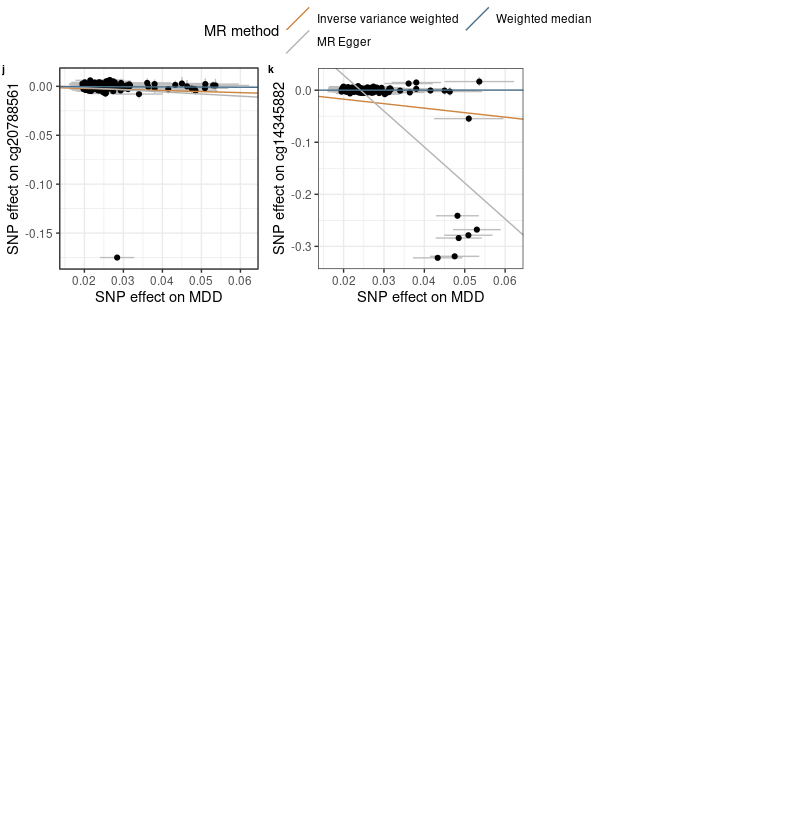


Figure S11. Scatter plot for MR of liability of depression to DNAm (j-k).

#####


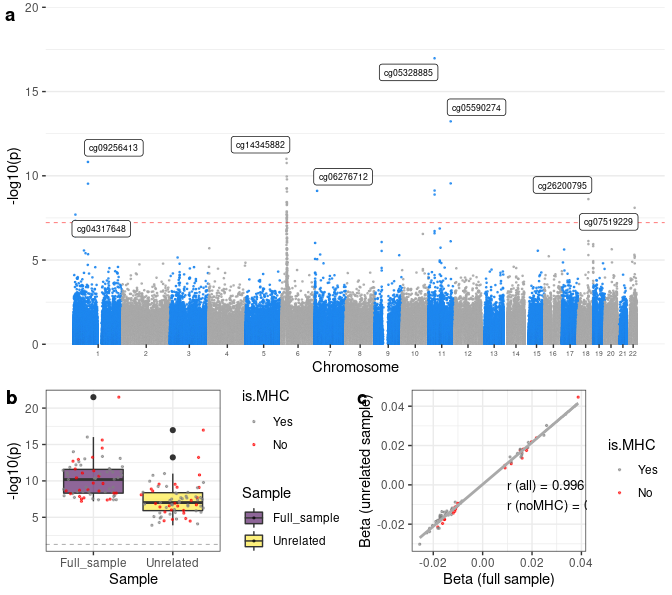


Figure S12. Supplementary MWAS on the unrelated sample in GS (N=6777). (a) Manhattan plot. X-axis represents the relative position of the probes in the genome. Y-axis represents -log10-transformed p values. The red dashed line represents the significance threshold for Bonferroni correction. CpGs that were significantly associated with depression-PRS at pT< 5×10_-8_ in the full-sample analysis are highlighted in yellow. (b) Box plot for the -log10-transformed p values for the analyses on the full sample versus the unrelated sample. CpG probes within the MHC region are marked in gray and those outside of the MHC region are marked in red. (c) Correlation between the effect sizes (beta) of the analyses conducted on the full sample and the unrelated sample. CpG probes within the MHC region are marked in gray and those outside of the MHC region are marked in red.

##### Table S1. Number of genetic variants used for calculating PRSs. pT = p threshold used for calculating PRS, N_SNP_ = total number of SNPs used for calculating PRS, N_noMHC_ = number of SNPs outside of the MHC region, N_MHC_ = number of SNPs within the MHC region.

| pT | N_SNP_ | N_noMHC_ | N_MHC_ |
| --- | --- | --- | --- |
| 5e-08 | 127 | 118 | 9 |
| 1e-05 | 615 | 602 | 13 |
| 1e-04 | 1457 | 1441 | 16 |
| 1e-03 | 4199 | 4176 | 23 |
| 0.01 | 14578 | 14530 | 48 |
| 0.05 | 38284 | 38185 | 99 |
| 0.1 | 58867 | 58742 | 125 |
| 0.5 | 151226 | 150977 | 249 |
| 1 | 199432 | 199113 | 319 |

##### Table S2. Association between PRSs and prevalence depression. PRS pT = p threshold used for selecting SNPs for calculating PRS.

| PRS pT | Beta | SE | p | R2 (%) |
| --- | --- | --- | --- | --- |
| 5e-08 | 0.114 | 0.033 | 5.52e-04 | 0.269 |
| 1e-05 | 0.157 | 0.032 | 1.28e-06 | 0.496 |
| 1e-04 | 0.208 | 0.032 | 4.80e-11 | 0.926 |
| 1e-03 | 0.236 | 0.032 | 1.40e-13 | 1.149 |
| 0.01 | 0.253 | 0.032 | 2.07e-15 | 1.332 |
| 0.05 | 0.323 | 0.033 | 3.02e-23 | 2.086 |
| 0.1 | 0.326 | 0.032 | 5.38e-24 | 2.161 |
| 0.5 | 0.294 | 0.032 | 8.20e-20 | 1.752 |
| 1 | 0.288 | 0.032 | 3.78e-19 | 1.688 |

##### Table S3. Association between PRSs and DNAm-estimated white-blood cell proportions.

|  | CD8T | | CD4T | | NK | | Bcell | | Mono | | Gran | |
| --- | --- | --- | --- | --- | --- | --- | --- | --- | --- | --- | --- | --- |
| PRS pT/depression | Beta | p | Beta | p | Beta | p | Beta | p | Beta | p | Beta | p |
| 0.00000005 | 0.78 | 0.079 | -1.316 | 0.054 | 0.238 | 0.594 | -0.162 | 0.63 | 0.42 | 0.113 | 0.039 | 0.97 |
| 0.000001 | 0.525 | 0.476 | -1.236 | 0.275 | -0.039 | 0.958 | -0.315 | 0.572 | 0.27 | 0.54 | 0.796 | 0.644 |
| 0.0001 | -1.012 | 0.652 | 0.383 | 0.912 | 2.06 | 0.36 | 0.429 | 0.8 | 0.95 | 0.478 | -2.811 | 0.593 |
| 0.001 | -7.709 | 0.085 | 6.729 | 0.329 | 1.34 | 0.765 | -1.442 | 0.67 | 2.603 | 0.331 | -1.521 | 0.885 |
| 0.01 | -11.225 | 0.264 | 15.929 | 0.303 | 8.285 | 0.411 | -5.734 | 0.451 | -0.025 | 0.997 | -7.229 | 0.759 |
| 0.05 | -10.981 | 0.564 | 19.472 | 0.506 | 6.817 | 0.721 | -6.856 | 0.634 | 2.248 | 0.843 | -10.701 | 0.81 |
| 0.1 | -20.887 | 0.415 | 16.367 | 0.678 | -1.843 | 0.943 | -11.93 | 0.538 | 5.41 | 0.724 | 12.882 | 0.83 |
| 0.2 | -16.002 | 0.648 | 34.044 | 0.528 | 6.382 | 0.856 | -12.215 | 0.645 | 0.219 | 0.992 | -12.428 | 0.879 |
| 0.5 | -20.493 | 0.704 | 49.481 | 0.552 | 5.893 | 0.913 | -22.849 | 0.576 | 2.459 | 0.939 | -14.491 | 0.909 |
| 1 | -31.487 | 0.654 | 46.742 | 0.666 | 5.893 | 0.933 | -32.108 | 0.546 | 2.006 | 0.962 | 8.954 | 0.957 |
| Depression status | -5.51e-04 | 0.634 | -0.002 | 0.226 | 1.48e-05 | 0.99 | -7.14e-04 | 0.422 | 7.97e-04 | 0.249 | 0.003 | 0.333 |

##### Table S4. Genomic inflation factor for discovery, adult replication (LBC 1921 + LBC 1936 + ALSPAC adults) and adolescent replication (ALSPAC adolescents) MWAS.

| p threshold | Discovery | Replication (adults) | Replication (adolescent) |
| --- | --- | --- | --- |
| 5e-08 | 0.97 | 0.987 | 1.006 |
| 1e-06 | 0.949 | 0.995 | 0.995 |
| 1e-04 | 0.968 | 0.97 | 1.04 |
| 1e-03 | 0.968 | 0.985 | 1.054 |
| 0.01 | 0.966 | 0.925 | 0.977 |
| 0.05 | 0.96 | 0.958 | 0.946 |
| 0.1 | 0.962 | 0.978 | 0.94 |
| 0.5 | 0.954 | 0.964 | 0.943 |
| 1 | 0.952 | 0.979 | 0.943 |

##### Table S5. Top CpG probes associated with PRS at pT = 5e-8. CHR=chromosome, BP=base pair position, SE = standard error, p.adj = Bonferroni-corrected p value, HetChiSq = Chi-square statistics for heterogeneity analysis, and HetPVal = p value for heterogeneity analysis.

| Location | CpG | CHR | BP | Beta | SE | p | p.adj | Direction | HetChiSq | HetPVal |
| --- | --- | --- | --- | --- | --- | --- | --- | --- | --- | --- |
| With MHC region | cg05328885 | 11 | 30943623 | 0.039 | 0.004 | 3.13e-22 | 2.41e-16 | ++ | 34.339 | 4.63e-09 |
|  | cg14345882 | 6 | 26364793 | -0.018 | 0.002 | 9.46e-17 | 7.28e-11 | -- | 1.233 | 0.267 |
|  | cg05590274 | 11 | 113262625 | 0.015 | 0.002 | 2.44e-16 | 1.88e-10 | ++ | 12.667 | 3.72e-04 |
|  | cg09256413 | 1 | 72566690 | -0.013 | 0.002 | 3.14e-15 | 2.42e-09 | -- | 7.462 | 0.006 |
|  | cg12740337 | 6 | 28058973 | -0.017 | 0.002 | 9.72e-15 | 7.48e-09 | -- | 0.282 | 0.595 |
|  | cg18844029 | 6 | 28885017 | -0.017 | 0.002 | 4.33e-14 | 3.33e-08 | -- | 2.126 | 0.145 |
|  | cg26200795 | 18 | 52895482 | 0.018 | 0.002 | 5.71e-14 | 4.40e-08 | ++ | 2.861 | 0.091 |
|  | cg26587870 | 6 | 27730563 | 0.026 | 0.004 | 8.05e-14 | 6.20e-08 | ++ | 2.26 | 0.133 |
|  | cg17849569 | 6 | 28058911 | -0.018 | 0.002 | 9.23e-14 | 7.10e-08 | -- | 0.04 | 0.842 |
|  | cg06627827 | 1 | 72898668 | -0.017 | 0.002 | 1.12e-13 | 8.62e-08 | -- | 2.151 | 0.142 |
| Without MHC region | cg05328885 | 11 | 30943623 | 0.039 | 0.004 | 3.13e-22 | 2.41e-16 | ++ | 34.339 | 4.63e-09 |
|  | cg05590274 | 11 | 113262625 | 0.015 | 0.002 | 2.44e-16 | 1.88e-10 | ++ | 12.667 | 3.72e-04 |
|  | cg09256413 | 1 | 72566690 | -0.013 | 0.002 | 3.14e-15 | 2.42e-09 | -- | 7.462 | 0.006 |
|  | cg26200795 | 18 | 52895482 | 0.018 | 0.002 | 5.71e-14 | 4.40e-08 | ++ | 2.861 | 0.091 |
|  | cg06627827 | 1 | 72898668 | -0.017 | 0.002 | 1.12e-13 | 8.62e-08 | -- | 2.151 | 0.142 |
|  | cg06941483 | 11 | 30874744 | 0.016 | 0.002 | 1.98e-13 | 1.52e-07 | ++ | 2.874 | 0.09 |
|  | cg06276712 | 7 | 12107011 | -0.011 | 0.002 | 2.29e-12 | 1.76e-06 | -- | 16.786 | 4.18e-05 |
|  | cg26647111 | 11 | 31128758 | -0.015 | 0.002 | 4.02e-12 | 3.10e-06 | -- | 4.767 | 0.029 |
|  | cg04317648 | 1 | 8485376 | 0.011 | 0.002 | 8.34e-12 | 6.42e-06 | ++ | 6.583 | 0.01 |
|  | cg14159747 | 11 | 113255604 | -0.016 | 0.002 | 2.25e-11 | 1.73e-05 | -- | 15.995 | 6.35e-05 |

##### Table S6. Results for discovery MR of DNAm to depression. N_SNP_ = Number of genetic instruments included in the analysis. IVW = inverse‐variance weighted, WM = weighted median, p.adj = FDR-corrected p value, p_Egger intercept_ = p value for Egger intercept evaluation, Q = Q statistics for heterogeneity, p_Q_ = p value for Q statistics.

|  | | IVW | | WM | | MR Egger | | QC measures | | | |
| --- | --- | --- | --- | --- | --- | --- | --- | --- | --- | --- | --- |
| Exposure | N_SNP_ | Beta | p.adj | Beta | p.adj | Beta | p.adj | Egger intercept | p_Egger intercept_ | Q | p_Q_ |
| cg00591421 | 7 | -0.016 | 0.014 | -0.013 | 0.101 | -0.012 | 0.643 | -0.001 | 0.779 | 3.97 | 0.681 |
| cg05590274 | 6 | 0.026 | 0.186 | 0.042 | 1.31e-04 | -0.027 | 0.703 | 0.016 | 0.372 | 36.44 | 7.77e-07 |
| cg06552810 | 8 | -0.02 | 0.004 | -0.015 | 5.10e-04 | -0.01 | 0.643 | -0.006 | 0.288 | 35.3 | 9.83e-06 |
| cg07519229 | 9 | -0.03 | 3.44e-08 | -0.027 | 6.29e-07 | -0.026 | 0.319 | -0.002 | 0.733 | 15.02 | 0.059 |
| cg14159747 | 8 | -0.029 | 4.42e-05 | -0.034 | 4.07e-07 | -0.034 | 0.319 | 0.002 | 0.749 | 13.32 | 0.065 |
| cg14345882 | 11 | -0.03 | 4.43e-07 | -0.026 | 2.07e-16 | -0.013 | 0.355 | -0.012 | 0.024 | 52.85 | 7.94e-08 |
| cg14844989 | 8 | -0.021 | 0.004 | -0.016 | 5.10e-04 | -0.004 | 0.703 | -0.01 | 0.123 | 35.55 | 8.81e-06 |
| cg19624444 | 7 | 0.026 | 6.08e-05 | 0.025 | 8.71e-10 | 0.028 | 0.319 | -0.001 | 0.797 | 16.23 | 0.013 |
| cg20788561 | 5 | -0.018 | 0.054 | -0.02 | 0.004 | -0.011 | 0.696 | -0.004 | 0.657 | 18.38 | 0.001 |
| cg23275840 | 5 | 0.04 | 1.34e-05 | 0.038 | 2.16e-10 | 0.017 | 0.643 | 0.009 | 0.291 | 13.47 | 0.009 |
| cg26647111 | 7 | -0.017 | 0.004 | -0.012 | 0.002 | -0.014 | 0.547 | -0.002 | 0.701 | 19.67 | 0.003 |

##### Table S7. Results for replication MR of DNAm to depression. N_SNP_ = Number of genetic instruments included in the analysis. IVW = inverse‐variance weighted, WM = weighted median, p.adj = FDR-corrected p value, p_Egger intercept_ = p value for Egger intercept evaluation, Q = Q statistics for heterogeneity, p_Q_ = p value for Q statistics.

|  | | IVW | | WM | | MR Egger | | QC measures | | | |
| --- | --- | --- | --- | --- | --- | --- | --- | --- | --- | --- | --- |
| Exposure | N_SNP_ | Beta | p.adj | Beta | p.adj | Beta | p.adj | Egger intercept | p_Egger intercept_ | Q | p_Q_ |
| cg00591421 | 13 | -0.092 | 5.48e-04 | -0.109 | 2.92e-04 | -0.163 | 0.011 | 0.005 | 0.081 | 13 | 0.37 |
| cg05590274 | 7 | 0.166 | 0.016 | 0.153 | 5.58e-05 | 0.047 | 0.732 | 0.01 | 0.341 | 31.2 | 2.29e-05 |
| cg06552810 | 13 | -0.048 | 0.015 | -0.032 | 0.003 | -0.025 | 0.483 | -0.004 | 0.364 | 50.1 | 1.36e-06 |
| cg07519229 | 15 | -0.134 | 2.83e-13 | -0.129 | 3.90e-08 | -0.185 | 0.004 | 0.004 | 0.145 | 18.5 | 0.186 |
| cg14159747 | 8 | -0.066 | 0.102 | -0.058 | 0.002 | -0.065 | 0.483 | -1.84e-04 | 0.983 | 37.7 | 3.48e-06 |
| cg14345882 | 22 | -0.136 | 1.15e-26 | -0.125 | 7.45e-30 | -0.117 | 0.004 | -0.004 | 0.475 | 69.3 | 4.49e-07 |
| cg14844989 | 12 | -0.054 | 0.017 | -0.036 | 0.004 | -0.028 | 0.483 | -0.004 | 0.404 | 49.7 | 7.14e-07 |
| cg19624444 | 10 | 0.081 | 1.67e-07 | 0.076 | 9.07e-10 | 0.087 | 0.015 | -0.001 | 0.77 | 18.8 | 0.027 |
| cg20788561 | 11 | -0.13 | 0.002 | -0.16 | 1.61e-04 | -0.109 | 0.385 | -0.002 | 0.777 | 20 | 0.029 |
| cg23275840 | 11 | 0.199 | 1.08e-23 | 0.192 | 4.32e-13 | 0.198 | 0.011 | 9.52e-05 | 0.981 | 10.4 | 0.408 |
| cg26647111 | 9 | -0.054 | 0.024 | -0.048 | 0.001 | -0.056 | 0.399 | 2.88e-04 | 0.963 | 25 | 0.002 |

##### Table S8. Results for multivariable MR of DNAm to depression.

| Exposure | N_SNP_ | Beta | SE | p |
| --- | --- | --- | --- | --- |
| cg26647111 | 1 | -1.359 | 1.268 | 0.283 |
| cg06552810 | 2 | -1.784 | 1.049 | 0.089 |
| cg19624444 | 5 | 0.077 | 0.049 | 0.114 |
| cg14345882 | 6 | 0.129 | 0.025 | 4.13e-07 |
| cg23275840 | 8 | -0.22 | 0.04 | 2.77e-08 |
| cg14844989 | 2 | 3.028 | 1.168 | 0.01 |
| cg07519229 | 3 | -0.072 | 0.061 | 0.232 |
| cg14159747 | 4 | -0.061 | 0.137 | 0.655 |

##### Table S9. Results for MR of liability of depression to DNAm. N_SNP_ = Number of genetic instruments included in the analysis. IVW = inverse‐variance weighted, WM = weighted median, p.adj = FDR-corrected p value, p_Egger intercept_ = p value for Egger intercept evaluation, Q = Q statistics for heterogeneity, p_Q_ = p value for Q statistics.

|  | | IVW | | WM | | MR Egger | | QC measures | | | |
| --- | --- | --- | --- | --- | --- | --- | --- | --- | --- | --- | --- |
| Outcome | N_SNP_ | Beta | p.adj | Beta | p.adj | Beta | p.adj | Egger intercept | p_Egger intercept_ | Q | p_Q_ |
| cg00591421 | 123 | -0.043 | 0.074 | -0.021 | 0.804 | -0.067 | 0.722 | 6.37e-04 | 0.776 | 383 | 1.06e-28 |
| cg05590274 | 122 | 0.027 | 0.169 | 0.008 | 0.882 | 0.011 | 0.965 | 4.38e-04 | 0.851 | 486 | 2.91e-45 |
| cg06552810 | 123 | -0.13 | 0.135 | 0.007 | 0.882 | 0.349 | 0.722 | -0.013 | 0.161 | 3626 | <1e-324 |
| cg06941483 | 122 | 0.108 | 0.135 | 0.017 | 0.804 | -0.28 | 0.722 | 0.01 | 0.176 | 3999 | <1e-324 |
| cg07519229 | 122 | -0.13 | 0.135 | 0.016 | 0.804 | 0.198 | 0.74 | -0.009 | 0.352 | 7239 | <1e-324 |
| cg09256413 | 122 | -0.086 | 0.003 | -0.024 | 0.393 | -0.251 | 0.12 | 0.004 | 0.125 | 1083 | 3.41e-154 |
| cg14159747 | 123 | -0.008 | 0.763 | 0.008 | 0.882 | -0.002 | 0.984 | -1.43e-04 | 0.963 | 521 | 9.68e-51 |
| cg14345882 | 123 | -0.864 | 8.85e-05 | 0.004 | 0.882 | -6.905 | 1.62e-22 | 0.167 | 9.73e-21 | 28358 | <1e-324 |
| cg14844989 | 123 | -0.109 | 0.135 | 0.005 | 0.882 | 0.313 | 0.722 | -0.011 | 0.164 | 3163 | <1e-324 |
| cg16996682 | 122 | 0.066 | 0.028 | 0.003 | 0.882 | 0.087 | 0.722 | -5.64e-04 | 0.845 | 608 | 5.04e-66 |
| cg19624444 | 122 | 0.266 | 0.135 | 0.004 | 0.882 | 0.333 | 0.747 | -0.002 | 0.925 | 11975 | <1e-324 |
| cg20788561 | 123 | -0.108 | 0.136 | -0.015 | 0.804 | -0.216 | 0.74 | 0.003 | 0.724 | 5307 | <1e-324 |
| cg23275840 | 122 | 0.147 | 0.135 | -0.004 | 0.882 | -0.187 | 0.74 | 0.009 | 0.327 | 9730 | <1e-324 |
| cg26272214 | 122 | 0.122 | 0.135 | 0.058 | 0.107 | 0.726 | 0.12 | -0.016 | 0.05 | 2799 | <1e-324 |
| cg26647111 | 123 | -0.096 | 0.135 | -0.004 | 0.882 | 0.214 | 0.722 | -0.008 | 0.203 | 2779 | <1e-324 |
